# The impact of post-operative sepsis on mortality after hospital discharge among elective surgical patients: a population-based cohort study

**DOI:** 10.1186/s13054-016-1596-7

**Published:** 2017-02-20

**Authors:** Lixin Ou, Jack Chen, Ken Hillman, Arthas Flabouris, Michael Parr, Hassan Assareh, Rinaldo Bellomo

**Affiliations:** 1 Simpson Centre for Health Services Research, South Western Sydney Clinical School, University of New South Wales, Sydney, New South Wales Australia; 2 Ingham Institute for Applied Medical Research, Liverpool, New South Wales Australia; 3 Intensive Care Unit, Royal Adelaide Hospital, Adelaide, South Australia Australia; 4 Faculty of Health Sciences, School of Medicine, University of Adelaide, Adelaide, South Australia Australia; 5 Intensive Care Unit, Liverpool Hospital, University of New South Wales, Sydney, New South Wales Australia; 6 Epidemiology and Health Analytics, South Western Sydney Local Health District, Sydney, New South Wales Australia; 7 School of Medicine, University of Melbourne, Parkville, Melbourne, Victoria Australia

## Abstract

**Background:**

Our aim in the present study was to assess the mortality impact of hospital-acquired post-operative sepsis up to 1 year after hospital discharge among adult non-short-stay elective surgical patients.

**Methods:**

We conducted a population-based, retrospective cohort study of all elective surgical patients admitted to 82 public acute hospitals between 1 January 2007 and 31 December 2012 in New South Wales, Australia. All adult elective surgical admission patients who stayed in hospital for ≥4 days and survived to discharge after post-operative sepsis were identified using the Admitted Patient Data Collection records linked with the Registry of Births, Deaths, and Marriages. We assessed post-discharge mortality rates at 30 days, 60 days, 90 days and 1 year and compared them with those of patients without post-operative sepsis.

**Results:**

We studied 144,503 survivors to discharge. Of these, 1857 (1.3%) had experienced post-operative sepsis. Their post-discharge mortality rates at 30 days, 60 days, 90 days and 1 year were 4.6%, 6.7%, 8.1% and 13.5% (vs 0.7%, 1.2%, 1.5% and 3.8% in the non-sepsis cohort), respectively (*P* < 0.0001 for all). After adjustment for patient and hospital characteristics, post-operative sepsis remained independently associated with a higher mortality risk (30-day mortality HR 2.75, 95% CI 2.14–3.53; 60-day mortality HR 2.45, 95% CI 1.94–3.10; 90-day mortality HR 2.31, 95% CI 1.85–2.87; 1-year mortality HR 1.71, 95% CI 1.46–2.00). Being older than 75 years of age (HR 3.50, 95% CI 1.56–7.87) and presence of severe/very severe co-morbidities as defined by Charlson co-morbidity index (severe vs normal HR 2.05, 95% CI 1.45–2.89; very severe vs normal HR 2.17, 95% CI 1.49–3.17) were the only other significant independent predictors of increased 1-year mortality.

**Conclusions:**

Among elective surgical patients, post-operative sepsis is independently associated with increased post-discharge mortality up to 1 year after hospital discharge. This risk is particularly high in the first month, in older age patients and in the presence of severe/very severe co-morbidities. This high-risk population can be targeted for interventions.

**Electronic supplementary material:**

The online version of this article (doi:10.1186/s13054-016-1596-7) contains supplementary material, which is available to authorized users.

## Background

Post-operative sepsis is a leading cause of multiple organ dysfunction and in-hospital mortality [1–3]. The U.S. Centers for Disease Control and Prevention reported that about 1 in 25 patients experience at least one healthcare-associated infection during hospitalisation [4]. Patients who are admitted with or who develop sepsis in hospital also have an increased risk of death following hospital discharge [5, 6]. Such sepsis-associated risk of death is higher than in the general population and remains higher for up to 5 years following hospital discharge [7, 8]. Moreover, long-term healthcare costs for sepsis survivors are higher. Researchers in a recent study reported that 42.7% of severe sepsis survivors were re-hospitalized within 90 days [9], incurring higher costs, especially in the first year after hospital discharge, when costs are approximately three times the costs in the following 2–3 years [10]. Given such high prevalence, significant risk of mortality, poor prognosis and high healthcare resource consumption, the U.S. Agency for Healthcare Research and Quality (AHRQ) has proposed ‘post-operative sepsis’ as a key patient safety indicator, aiming to monitor potentially preventable surgical complications among elective surgical patients without serious medical conditions at admission [11]. Thus, the indicator ‘post-operative sepsis’ was developed after a comprehensive literature review; analysis of International Classification of Diseases, Ninth Revision, Clinical Modification (ICD-9-CM), codes; review by a clinician panel; implementation of risk adjustment; and performing empirical analyses. This quality indicator has been widely used in the United States to measure aspects of patient safety and quality and to monitor the impact of quality improvement initiatives [3, 11–14].

Despite the importance of sepsis, most studies in which investigators have examined the long-term outcomes of sepsis survivors have been limited to a single institution [10, 15–17], or to patients specifically admitted via emergency rooms [18], or to intensive care units (ICUs) [7, 19, 20]. Most of these long-term outcome studies have included all sources of sepsis, such as a mix of community- or hospital-acquired sepsis, as well as a mix of medical and surgical patients [5]. In contrast, the few studies in which researchers have examined 30-day or 1-year post-discharge mortality among elective surgical patients with post-operative sepsis have narrowly targeted specific patient groups (e.g., patients with cancer or elderly patients aged ≥65 years) and surgery types (e.g., digestive or abdominal aortic aneurysm [AAA] surgery), limiting their generalisability [21, 22].

Accordingly, we conducted a population-based study of long-term mortality among patients with post-operative sepsis up to 1 year after discharge, and we compared post-discharge mortality and its associated risk factors with those of elective surgical patients without post-operative sepsis admitted to all public acute hospitals in the state of New South Wales (NSW), Australia. We targeted those patients who stayed in hospital beyond 3 days and survived to discharge, and we aimed to test the hypothesis that patients with post-operative sepsis who are discharged alive from hospital have a higher risk of death, even at 1 year after their index admission. We also explored the hypothesis that post-operative sepsis in these patients is an independent predictor of death and that other risk factors can be identified that are associated with such increased risk.

## Methods

### Data source and study population

We performed a population-based retrospective cohort study using NSW administrative data derived from the Admitted Patient Data Collection (APDC) records. The APDC includes information on patient demographics, medical conditions and procedures, hospital characteristics, and separations (discharges, transfers and deaths) from all hospitals in NSW. The medical records for each episode of care in the APDC were assigned codes based on the International Statistical Classification of Diseases and Related Health Problems, Tenth Revision, Australian Modification (ICD-10-AM) [23]. Each hospital has certified and trained coders who follow standardised procedures to generate these codes from information in medical records.

**Fig. 1:**
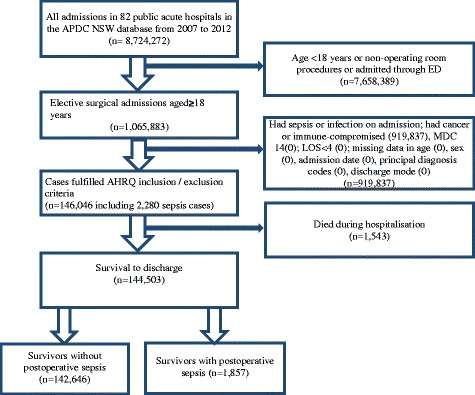
Flowchart illustrating the derivation of the study population. *AHRQ* Agency for Healthcare Research and Quality, *APDC* Admitted Patient Data Collection, *ED* Emergency department, *LOS* Length of stay, *MDC* Major Diagnostic Categories

The study included adult elective surgical patients who were admitted to 82 NSW public acute hospitals between 1 January 2007 and 31 December 2012 and survived to discharge. We identified our study population on the basis of selection criteria developed by the AHRQ for post-operative sepsis. According to the AHRQ selection criteria, we included all elective surgical patients (aged ≥18 years) who had any primarily performed procedures with operating room procedure codes and not admitted through the emergency department and who did not have a short hospital stay (Fig. 1). We excluded those patients who fulfilled any one of the following exclusion criteria:

- A principal diagnosis on admission that was sepsis or infection (see Additional file 1: Appendix 1; Additional file 2: Appendix 2)
- Any ICD-10-AM diagnosis codes for cancer (see Additional file 2: Appendix 2)
- Any ICD-10-AM diagnosis codes or any ICD-10-AM procedure codes for immunocompromised state (see Additional file 2: Appendix 2)
- Major Diagnostic Categories 14 (pregnancy, childbirth and puerperium)
- A stay in hospital of less than 4 days
- Missing data on discharge status, sex, age, year or principal diagnosis (There were no missing values for these variables among the elective surgical admissions aged ≥18 years included in our study.)

Among the selected population, cases with post-operative sepsis were identified by ICD-10-AM diagnosis codes (see Additional file 1: Appendix 1). Because of the difference in coding systems used in the United States (ICD-9-CM) and Australia (ICD-10-AM), all diagnoses and procedure codes in the AHRQ definition were translated to ICD-10-AM codes according to the Organisation for Economic Co-operation and Development (OECD) technical manual for patient safety indicators [24]. We derived outcome variables of post-operative sepsis using 54 non-principal diagnostic fields in the medical record by ICD-10-AM codes matched from the OECD manual (see Additional file 1: Appendix 1). We then classified study population into sepsis and non-sepsis cohorts (Fig. 1).

The selected data were linked to the NSW Registry of Births, Deaths, and Marriages (RBDM) through the Centre of Health Record Linkage, NSW Ministry of Health, to derive survival status within 1 year after discharge. We excluded those patients whose survival status was not available up to 1 year after discharge because the RBDM data obtained in our study spanned only from 1 January 2007 to 28 March 2014. As a result, the study period was presented as admission years between 2007 and 2012.

### Patient demographic and hospital characteristics

Patient demographic information included age, sex, country of birth, marital status, severity of illness/co-morbidity, and advantaged and disadvantaged Socio-Economic Indexes for Areas (SEIFA) scores [25] (categorised into four classes, from first quartile = most disadvantaged areas to fourth quartile = most advantaged areas) representing patient socio-economic status. Severity of illness/co-morbidity was defined by the Charlson co-morbidity index score based on the ICD-10 coding scheme [26]. We classified the severity of illness/co-morbidity into four categories: normal (index score = 0), moderate (index score = 1), severe (index score = 2) and very severe (index score ≥3). Because the AHRQ selection criteria excluded patients with any cancer or immunocompromised state, three of the Charlson co-morbidities—any malignancy (including leukaemia and lymphoma), metastatic solid tumour and AIDS/HIV—were excluded. Thus, the co-morbidities in this study encompassed only 14 of the Charlson co-morbidity index-defined co-morbidities: myocardial infarction, congestive heart failure, peripheral vascular disease, cerebrovascular disease, dementia, chronic pulmonary disease, rheumatologic disease, peptic ulcer disease, mild liver disease, diabetes without chronic complications, diabetes with chronic complications, hemiplegia or paraplegia, renal disease, and moderate or severe liver disease.

Hospital characteristics, including the location (metropolitan, rural and regional NSW) as well as peer group classification: (A1 = principal referral, usually teaching hospitals; A3 = ungrouped acute; B = major metropolitan and non-metropolitan; C1 = district group 1; C2 = district group 2). Peer hospital groups were divided into those of similar type and size, ranging from treating 25,000 or more acute case-mix-weighted separations per annum in the principal referral group through to treating ≥2000 or more (but <5000) acute case-mix-weighted separations per annum in district group 2 [27]. Using appropriate procedure codes from ICD-10-AM (Additional file 3: Appendix 3) [24], we defined six groups of major surgical procedures: coronary artery bypass graft (CABG), abdominal surgery, endovascular aneurysm repair, total hip replacement, total knee replacement and other surgical procedures.

### Study outcomes

The primary outcome was post-hospital discharge mortality at 30 days, 60 days, 90 days and 1 year. We used the date of death and the date of discharge to define the length of survival. Post-discharge mortality in the sepsis cases was calculated as the number of deaths in the group at 30 days, 60 days, 90 days and 1 year after the discharge date divided by the number of sepsis cases, respectively. A similar calculation of post-discharge mortality was performed in the non-sepsis group.

### Statistical analysis

Baseline characteristics were compared between the non-sepsis and sepsis cohorts by unpaired *t* test and the Rao-Scott chi-square test. To understand changes in 1-year mortality over time, we assessed the crude linear trend for the outcome variables after excluding a possible quadratic effect using the study year as a continuous variable and employing Poisson mixed models. Cumulative survival outcomes across the non-sepsis and sepsis cohorts were estimated first by calculating the Kaplan-Meier survival functions and using log-rank tests to detect differences between the two cohorts up to 1 year after discharge for differences in age-, sex- and co-morbidity-specific mortality rates in the two cohorts. We then estimated the risk difference of death at 30 days, 60 days, 90 days and 1 year between the two cohorts using Cox proportional hazards models to control for the other confounding factors, which included patient demographics (baseline age, sex, country of birth, marital status, severity of illness/co-morbidity and SEIFA score), hospital characteristics (location and peer groups), major surgical procedures, length of hospital stay and the year of admission.

We conducted a sensitivity analysis using 1:1 nearest-neighbour matching (NNM) (based on Euclidean matrix) as recommended on the basis of recent research [28], in comparison with the popular propensity score matching (PSM) (1:1; based on logistic regression). For NNM, the matching was based on admission year, age, sex, country of birth, marital status, severity of illness/co-morbidity, socio-economic status (quartile of SEIFA score), location of the hospital (rural/regional vs others), hospital peer group, surgery type and length of stay. (The exact matching was done on the basis of age group, country of birth and co-morbidity.) The estimation of treatment effect was based on robust variance estimator and adjusted for age and length of stay. For PSM, the same matching variables were used, except that there were no exact matching groups and no adjustment for the continuous variables of age and length of stay. For both NNM and PSM, a caliper of 0.05 was employed, and a check for overlap of both baseline distribution and matched samples was done. Both graphic plots and standardised statistical summary and tests were employed wherever appropriate to ensure the balance of matched samples. We present the results from both NNM and PSM in Additional file 4: Appendix 4.

We took into account hospital cluster effect within these models using a robust cluster variance estimator. The risk of death is presented as an HR, and 95% CIs were calculated around the estimated HRs. A *P* value of 0.05 was considered as indicative of statistical significance for sepsis and non-sepsis cohorts, and 95% CIs are presented where appropriate. All analyses were performed using STATA 14 software (StataCorp, College Station, TX, USA). This study was approved by the NSW Population & Health Services Research Ethics Committee.

## Results

**Fig. 2:**
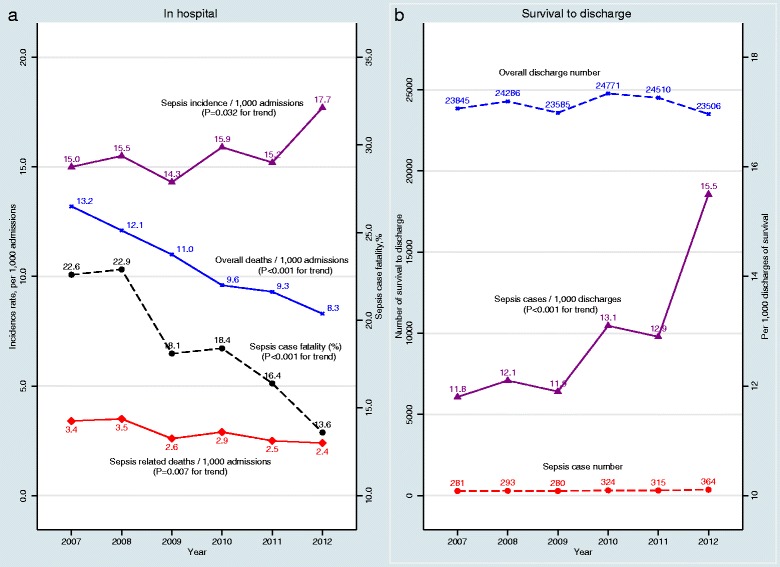
Trends in the incidence rate of post-operative sepsis and sepsis-related mortality in hospitals (**a**) and the number of survival to discharge (**b**), 2007–2012

A total of 146,046 elective surgical admission patients between 2007 and 2012 fulfilled the study selection criteria, and 2279 (15.6 per 1000 cases) developed post-operative sepsis. Of the 144,503 patients who survived to hospital discharge, 1857 (12.9 per 1000 cases) were hospital survivors after post-operative sepsis (Fig. 1, Table 1). The number of elective surgical patients who survived to discharge was similar in each admission year. However, the incidence of post-operative sepsis increased over time, whereas both the overall mortality rate and the mortality rate of those with sepsis declined (Fig. 2) over time. Accordingly, the proportion of post-operative sepsis survivors among all elective surgical patients increased from 11.8 per 1000 cases in 2007 to 15.5 per 1000 cases in 2012 (31.4% increase; *P* < 0.001 for trend) (Fig. 2). Compared with the non-sepsis cohort, patients with sepsis were older, more likely to be male, more likely to have severe/very severe illness, and more likely to be admitted to a hospital in a metropolitan area or to a principal referral hospital (Table 1). Almost half of the patients in the sepsis group and one-third in the non-sepsis group underwent CABG or abdominal surgery. The length of hospital stay for patients with sepsis was much longer than that of the non-septic patients (33.4 days vs 9.1 days on average, *P* < 0.001).

**Table 1:** Baseline patient demographic and hospital characteristics according to presence or absence of post-operative sepsis^a^ (*n* = 144,503)

Characteristics	Non-sepsis	Sepsis	IR of sepsis
Total	142,646	1857	12.9
Year of admission			
2007	23,564 (16.5%)	281 (15.1%)	11.8
2008	23,993 (16.8%)	293 (15.8%)	12.1
2009	23,305 (16.3%)	280 (15.1%)	11.9
2010	24,447 (17.1%)	324 (17.5%)	13.1
2011	24,195 (17.0%)	315 (17.0%)	12.9
2012	23,142 (16.2%)	364 (19.6%)	15.5
Hospitalisation in preceding year			
2007	N/A	N/A	
2008	2275/23,993 (9.5%)	35/293 (12.0%)	
2009	2734/23,305 (11.7%)	40/280 (14.3%)	
2010	3344/24,447 (13.7%)	46/324 (14.2%)	
2011	3771/24,195 (15.6%)	54/315 (17.1%)	
2012	3889/23,142 (16.8%)	64/364 (17.6%)	
Age			
≥18 years to <35 years	8838 (6.2%)	106 (5.7%)	11.9
≥35 years to <55 years	28,015 (19.6%)	348 (18.7%)	12.3
≥55 years to <75 years	64,382 (45.1%)	814 (43.8%)	12.5
≥75 years	41,411 (29.0%)	589 (31.7%)*	14
Mean ± SD	63.8 ± 16.3	64.8 ± 16.3**	
Sex			
Male	67,011 (47.0%)	1147 (61.8%)**	16.8
Female	75,635 (53.0%)	710 (38.2%)**	9.3
Country of birth			
Australia and New Zealand	98,787 (69.3%)	1249 (67.3%)	12.5
United Kingdom, United States and Canada	10,205 (7.2%)	111 (6.0%)*	10.8
Non-English-speaking Europe	15,949 (11.2%)	220 (11.8%)	13.6
North Africa	5564 (3.9%)	78 (4.2%)	13.8
Asia	6,922 (4.9%)	93 (5.0%)	13.3
Others	4,222 (3.0%)	77 (4.1%)**	17.9
Unknown	997 (0.7%)	29 (1.6%)**	28.3
Marital status			
Married	77,810 (54.6%)	1005 (54.1%)	12.8
Single	62,745 (44.0%)	805 (43.3%)	12.7
Unknown	1923 (1.3%)	47 (2.5%)**	23.9
Severity of illness/co-morbidity			
Normal	116,031 (81.3%)	969 (52.2%)**	8.3
Moderate	16,509 (11.6%)	322 (17.3%)**	19.1
Severe	5700 (4.0%)	213 (11.5%)**	36.0
Very severe	4406 (3.1%)	353 (19.0%)**	74.2
SEIFA scores			
First quartile (most disadvantaged)	48,634 (34.1%)	590 (31.8%)*	12
Second quartile	41,195 (28.9%)	568 (30.6%)	13.6
Third quartile	31,862 (22.3%)	408 (22.0%)	12.6
Fourth quartile (most advantaged)	19,888 (13.9%)	267 (14.4%)	13.2
Unknown	1067 (0.7%)	24 (1.3%)**	22
Local health district of facilities			
Metropolitan	91,018 (63.8%)	1336 (71.9%)**	14.5
Rural	44,902 (31.5%)	420 (22.6%)**	9.3
Unknown	6726 (4.7%)	101 (5.4%)	14.8
Peer hospital group			
Principal referral	85,108 (59.7%)	1358 (73.1%)**	15.7
Ungrouped acute	2418 (1.7%)	6 (0.3%)**	2.5
Major metropolitan and non-metropolitan	44,850 (31.4%)	444 (23.9%)**	9.8
District groups 1 and 2	10,270 (7.2%)	49 (2.6%)**	4.7
Major surgical procedure			
CABG	8994 (6.3%)	186 (10.0%)**	20.3
Abdominal surgery	33,506 (23.5%)	649 (35.0%)**	19.0
EVAR	1458 (1.0%)	20 (1.1%)	13.5
Total hip replacement	13,632 (9.6%)	45 (2.4%)**	3.3
Total knee replacement	22,598 (15.8%)	57 (3.1%)**	2.5
Other	62,458 (43.8%)	900 (48.5%)**	14.2
Length of stay			
Mean ± SD	9.1 ± 9.6	33.4 ± 31.6	
Median (IQR)	6 (5–10)	24 (14–42)	

*Abbreviations: IR* Incidence rate of sepsis reported per 1000 admissions, *SEIFA* Socio-Economic Indexes for Areas, *CABG* Coronary artery bypass graft, *EVAR* Endovascular aneurysm repair

^a^The two cohorts were compared by *t* test for continuous values or chi-square test for dichotomous values

*P ≤ 0.05 for difference between cohorts

**P ≤ 0.01 for difference between cohorts

### Mortality at 30 days, 60 days, 90 days and 1 year post-discharge

The unadjusted post-discharge mortality rates at 30 days, 60 days, 90 days and 1 year in the sepsis cohort were 4.6%, 6.7%, 8.1% and 13.5%, respectively (vs 0.7%, 1.2%, 1.5% and 3.8% in the non-sepsis cohort, respectively; *P* < 0.0001) (Table 2) with a proportionally strong increase in risk in the first 30 days.

**Table 2:** Post-discharge mortality and adjusted HRs at 30 days, 60 days, 90 days and 1 year between the two cohorts (pooled 2007–2012 data, *n* = 144,503)

Days after discharge	Mortality, %			HR (95% CI)	
	Non-sepsis (*n* = 142,646)	Sepsis (*n* = 1857)	*P* value	Unadjusted	Adjusted
30 days	0.7	4.6	<0.001	6.64 (5.23–8.44)	2.75 (2.14–3.53)
60 days	1.2	6.7	<0.001	5.90 (4.67–7.45)	2.45 (1.94–3.10)
90 days	1.5	8.1	<0.001	5.52 (4.40–6.91)	2.31 (1.85–2.87)
1 year	3.8	13.5	<0.001	3.79 (3.25–4.42)	1.71 (1.46–2.00)

*Note:* The adjusted HRs were derived from Poisson mixed models adjusted for age, sex, country of birth, marital status, severity of illness/co-morbidity, Socio-Economic Indexes for Areas (SEIFA), hospital location, peer hospital group, major surgical procedures and length of stay

**Fig. 3:**
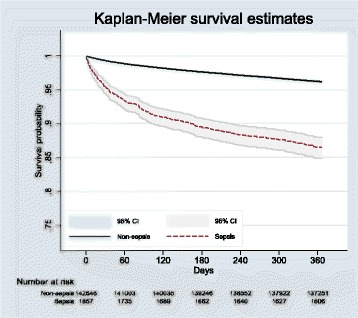
Overall Kaplan-Meier survival curve for elective surgical patients who survived to discharge, stratified by the presence or absence of post-operative sepsis

**Fig. 4:**
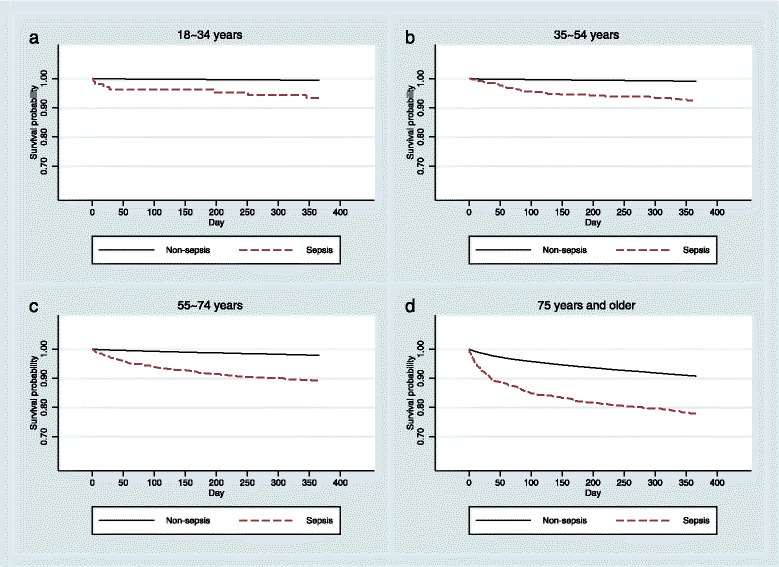
Age-specific Kaplan-Meier survival curves for elective surgical patients who survived to discharge, stratified by the presence or absence of post-operative sepsis. **a** Patients aged 18–34 years. **b** Patients aged 35–54 years. **c** Patients aged 55–74 years. **d** Patients aged 75 years and older

Kaplan-Meier survival analysis confirmed a significantly reduced survival at 1 year post-discharge in patients with sepsis (86.5% vs 96.2%; *P* < 0.001 by log-rank test) (Fig. 3), with the greatest rate of decline occurring within the first 30 days post-discharge. Although decreased survival in patients with sepsis was observed across all four age groups (*P* < 0.001) (Fig. 4), the effect was most pronounced among patients aged 75 years or older (Fig. 4d). After adjustment for patient and hospital characteristics, the risk of mortality was consistently higher at all time points in the sepsis cohort than in the non-sepsis cohort (Table 2). Both NNM and PSM approaches showed a consistent, elevated, significant risk of post-discharge mortality (at 30 days, 60 days and 1 year) (see Additional file 4: Appendix 4). These analyses showed that the absolute risk of death at 1 year post-discharge in the sepsis cohort was 5.4% higher than in the non-septic cohort.

### Multivariate analysis of 1-year mortality and its trends

The 1-year post-discharge mortality did not change significantly over the study period (2007–2012) in the non-sepsis and sepsis cohorts (Table 3). The Cox proportional hazards models displayed different patterns of the risk factors predicting 1-year mortality in the two cohorts. In the non-sepsis cohort, individual factors, including advanced age, male sex, place of birth, marital status, hospital peer group, surgical type and number of co-morbidities, predicted increasing risk of death. In the sepsis cohort, only older age (75 years or older; HR 3.50, 95% CI 1.56–7.87), hospital peer group, surgical type and pre-exiting severe/very severe co-morbidities (severe HR 2.05, 95% CI 1.45–2.89; very severe HR 2.17, 95% CI 1.49–3.17) carried an increased risk of death at 1 year after discharge. Older age and severity of illness/co-morbidity were two independent risk factors that increased the risk of death in both the sepsis and non-sepsis cohorts.

**Table 3:** Adjusted Cox proportional hazards models predicting 1-year post-discharge mortality

Characteristics	Non-sepsis (*n* = 142,646)		Sepsis (*n* = 1857)	
	Mortality, %	HR (95% CI)	Mortality, %	HR (95% CI)
Year of admission^†^				
2007	4.1	1.00	16.7	1.00
2008	4.1	1.03 (0.95–1.12)	10.9	0.70 (0.43–1.14)
2009	3.9	1.03 (0.94–1.12)	11.8	0.76 (0.48–1.21)
2010	3.7	1.05 (0.92–1.19)	16.1	1.04 (0.76–1.43)
2011	3.7	1.05 (0.96–1.15)	14	0.88 (0.60–1.31)
2012	3.5	0.98 (0.89–1.09)	11.8	0.78 (0.48–1.28)
Age groups				
≥18 years to <35 years	0.5	1.00	6.6	1.00
≥35 years to <55 years	0.9	1.99** (1.35–2.93)	7.5	1.10 (0.40–3.04)
≥55 years to <75 years	2.1	5.46** (3.77–7.92)	10.8	1.60 (0.76–3.38)
≥75 years	9.2	20.0** (13.5–29.6)	22.1	3.50** (1.56–7.87)
Sex				
Male	4.3	1.00	13.4	1.00
Female	3.4	0.76** (0.71–0.82)	13.7	1.05 (0.81–1.37)
Country of birth				
Australia and New Zealand	4.1	1.00	13.9	1.00
United Kingdom, United States and Canada	4.4	0.96 (0.88–1.05)	11.7	0.66 (0.36–1.19)
Non-English-speaking Europe	4.1	0.83** (0.74–0.93)	17.3	0.97 (0.69–1.36)
North Africa	1.5	0.50** (0.44–0.58)	7.7	0.52 (0.18–1.53)
Asia	1.9	0.68** (0.54–0.85)	10.8	0.80 (0.37–1.72)
Others	1.7	0.66** (0.53–0.83)	6.5	0.47 (0.18–1.21)
Unknown	5.6	1.02 (0.81–1.28)	17.2	1.45 (0.76–2.78)
Marital status				
Married	2.9	1.00	12.4	1.00
Single	4.8	1.37** (1.28–1.46)	14.9	1.10 (0.89–1.36)
Unknown	6.4	1.67** (1.37–2.03)	12.8	0.93 (0.39–2.24)
Severity of illness/co-morbidity				
Normal	2.5	1.00	9.2	1.00
Moderate	7.5	2.04** (1.88–2.21)	9.9	0.96 (0.61–1.50)
Severe	10.1	2.34** (2.01–2.72)	20.2	2.05** (1.45–2.89)
Very severe	15.5	3.34** (2.99–3.72)	24.6	2.17** (1.49–3.17)
SEIFA quartiles				
First quartile (most disadvantaged)	4.0	1.00	14.9	1.00
Second quartile	3.7	0.93 (0.85–1.00)	13.2	0.91 (0.65–1.26)
Third quartile	3.5	0.94 (0.83–1.06)	11.8	0.71* (0.52–0.97)
Fourth quartile (most advantaged)	4.3	0.99 (0.80–1.23)	13.1	0.68 (0.35–1.32)
Unknown	2.4	0.64* (0.41–1.00)	20.8	1.59 (0.90–2.84)
Local health district of facility				
Metropolitan	3.7	1.00	13.8	1.00
Rural and regional NSW	4.3	1.06 (0.88–1.27)	12.6	0.81 (0.60–1.10)
Others	3.1	0.72** (0.64–0.81)	12.9	1.08 (0.70–1.67)
Peer hospital group				
Principal referral group	3.9	1.00	12.4	1.00
Ungrouped acute	2.2	0.59** (0.44–0.80)	0	–
Major metropolitan and non-metropolitan	4	1.20 (1.00–1.45)	17.1	1.53* (1.10–2.11)
District groups 1 and 2	2.7	1.10 (0.85–1.44)	12.2	1.04 (0.40–2.73)
Major surgical procedure				
CABG	2.0	1.00	7.0	1.00
Abdominal surgery	2.8	1.91** (1.50–2.43)	8.3	1.25 (0.61–2.56)
EVAR	7.3	2.40** (1.92–3.00)	25.0	3.79** (1.39–10.3)
Total hip replacement	1.5	0.80 (0.62–1.03)	11.1	1.51 (0.47–4.78)
Total knee replacement	0.8	0.46** (0.36–0.59)	5.3	0.68 (0.19–2.42)
Other	6.1	3.13** (2.50–3.91)	19.0	2.69** (1.38–5.22)
Length of stay		1.01** (1.01–1.01)		1.00** (1.00–1.01)

*Abbreviations: SEIFA* Socio-Economic Indexes for Areas, *CABG* Coronary artery bypass graft, *EVAR* Endovascular aneurysm repair, *NSW* New South Wales

^†^ *P* value for trend year; *P* < 0.001 for non-sepsis group; *P* = 0.588 for sepsis group

*P ≤ 0.05

**P ≤ 0.01

The magnitude of risk of age on 1-year post-discharge mortality in sepsis cohort (HR 3.50 for age ≥75 years) was smaller than that in the non-sepsis cohort (HR 20.0). The risk of severity of illness/co-morbidity on death was also lower in the sepsis cohort (moderate vs normal HR 0.96, 95% CI 0.61–1.50; non-significant) than that in the non-sepsis cohort (HR 2.04, 95% CI 1.88–2.21).

## Discussion

### Key findings

We found that, among hospital survivors of elective surgery, the proportion experiencing post-operative sepsis increased 34% over the study period, although overall elective surgical activity was stable. Importantly, we found that survivors who had experienced post-operative sepsis had substantially higher post-discharge 1-year mortality (13.5%) than patients without post-operative sepsis (3.8%). This effect was more pronounced in the elderly, in those with co-morbidities and within the first 30 days post-discharge. Although age, sex, country of birth, marital status and co-morbidity strongly predicted the risk of long-term outcomes in the non-sepsis cohort, only advanced age and severe/very severe co-morbidities were independent predictors of 1-year mortality in the sepsis cohort. Finally, there was no significant change in 1-year post-discharge mortality for sepsis over the 6-year study period.

### Relationship to previous studies

Previous studies have shown that post-discharge 1-year mortality in patients with the general diagnosis of sepsis ranged from 21.5% for those admitted through emergency departments [15] to 71.9% for those discharged from an ICU [17]. Such values are much higher than our reported rate of 13.5% [18, 20]. This is not surprising, because elective surgical patients have a much lower overall mortality [29]. However, our point estimate was in line with recent systematic review results (14.0–18.0%) based on 43 studies [30].

Studies of long-term mortality related to sepsis in non-short-stay patients having elective surgery are few and have been focused on either specific types of surgery such as endovascular surgery or open abdominal aneurysm repair [21], or patients with cancer having gastrointestinal surgery [22]. In contrast, we studied all elective surgical patients, thereby providing the first estimate of risk on the broader population having elective surgery. Moreover, we excluded patients with a principal diagnosis of sepsis or infection, as well as those with cancer or who were immunocompromised. Thus, our study population was more likely to reflect patients for whom post-operative sepsis was potentially preventable. Nonetheless, the reported post-hospital discharge 30-day and 90-day mortality for patients undergoing elective open surgery and endovascular repair of non-ruptured AAAs, using the AHRQ definition of post-operative sepsis, were similar to ours [21]. Finally, we identified post-operative sepsis on the basis of the AHRQ definition, which differs slightly from the definitions used in other studies. None of the 11 studies [8, 31–40] which used administrative data in studying sepsis and its outcomes adopted exactly the same definition and research questions as we did in our present study, which made explicit comparisons impossible.

Our study findings of consistently elevated risk of post-discharge mortality up to 1 year among the sepsis survivors compared with the non-sepsis cohort were in contrast with a recently published systematic review [30] in which the authors did not consistently observe a causal relationship between sepsis and post-discharge mortality. Such consistently elevated risks were presented according to three approaches adopted in our study: a multivariate Cox regression model, NNM and PSM. Of these approaches, NNM showed the most significant effect. The reasons why our study showed such a positive link may be due to the facts that our study sample included only elective surgical patients with specific inclusion criteria; that our study sample was more homogeneous and the control cohort was more comparable; and that our study included more recent data (January 2007 through December 2012) than most of the study data included in the review, which was prior to 2005. However, our study also showed results consistent with those of the review in that age and co-morbidities were independent predictors of mortality among sepsis survivors.

Our detailed analyses confirmed that the 1-year post-discharge mortality gap between septic and non-septic elective surgical patients was particularly pronounced among the elderly and among those patients with co-morbidities [17, 41]. Researchers in previous studies reported similar in-hospital mortality rates between female and male surgical patients who developed severe sepsis or septic shock and those admitted to an ICU [42]. We also found similar 1-year post-discharge mortality rates between male and female patients in the septic cohort.

### Study implications

Our study has several clinical and policy implications. The data show that more than one in eight non-short-stay surgical patients who develop post-operative septic patients will die within 1 year. Being elective hospital admissions, some of these deaths may be preventable. As older patients with significant co-morbidities appear to be more at risk, our study implies such patients warrant closer assessment and targeted strategies to reduce the risk of post-operative sepsis. Our study also implies that assessing the impact of post-operative sepsis using survival at hospital discharge is flawed [12, 43]. Our finding that the greatest rate of decline in survival is within the first 30-day period after discharge implies that this immediate post-discharge period has the greatest potential as the key time frame for intervention.

It is worth noting that the incidence of post-operative sepsis had increased while the case fatality declined over the 6-year (2007–2012 inclusive) period, showing that the overall incidence rate of sepsis-related deaths in hospital barely changed between 2009 and 2012. We cannot offer a definitive answer on whether such an increase in the sepsis incidence rate was due to better coding or a real rise in the incidence. However, our results also show that the 1-year post-discharge mortality among sepsis survivors did not change significantly over the same period. This may imply that the increased coding of sepsis is unlikely to be the case for the observed increased incidence rate because it is likely that increased coding practice may pick up mostly less severe sepsis cases. As a result, the increased incidence of sepsis will lead to an increased number of sepsis survivors in better health and reduced 1-year post-discharge mortality over that time. On the contrary, our results showed a flat 1-year post-discharge mortality over that time, suggesting that more patients with sepsis were discharged alive but died within 1 year (most likely within 30 days) during the study period. Such results highlight the importance of developing new policy initiatives in providing better coordinated care for these patients and managing this shifted care burden.

### Strengths and limitations

This study has several strengths. To the best of our best knowledge, this is the largest population-based epidemiological study to provide evidence of an increased risk of post-discharge mortality for non-short-stay elective surgical patients with post-operative sepsis. Our study is also the first to demonstrate a persistently high risk of death among those sepsis survivors over a 12-month post-operative period. Our findings also identify more vulnerable patient subgroups, potentially providing clinical and policy-relevant information that could be used for benchmarking and translational interventions.

Our study also carries some limitations. First, we excluded patients with cancer and those who were immunologically compromised or those with a short hospital stay. In this study, we intended to target those patients whose sepsis was likely to be acquired through the exogenous factors which lent themselves to being more likely to be preventable. Moreover, these patients are also more likely to have benefited from the prompt clinical intervention and rescue, even when they developed sepsis during their hospitalization (e.g., the ‘failure-to-rescue’ definition of the AHRQ). However, we also acknowledge the fact that, in some cancer or immunocompromised patients who developed sepsis, their sepsis may also, in part, have been preventable, and they may have been rescued. Thus, we cannot comment on the incidence and implications of post-operative sepsis in such patients. However, our goal was to assess preventable post-operative sepsis in the presence of the smallest possible number of confounders and after excluding very low-risk patients. Second, despite the use of professional and certified coders to extract chart data, the absolute accuracy of such data extraction cannot be guaranteed. However, all large database analyses must depend on such coding, and the administrative data extracted by certified professional coders based on standardised guidelines at each hospital is unlikely to carry systematic bias.

Third, there may be a very small number of patients who were discharged to and died at another hospital, which could lead to an underestimation of in-hospital mortality. Despite the fact that we excluded those elective surgical patients who were principally diagnosed with sepsis or infection on admission, given the possibility of miscoding and misdiagnosis, further studies may be needed to provide an estimate of the extent that some of these septic cases may represent community-acquired sepsis. Furthermore, we did not have data on the time between surgery and post-operative sepsis events, surgical sites, and post-operative sepsis, and thus we could not make a clear differentiation between the surgical site infections vs non-surgical site infections among sepsis cohorts. Further designated studies are needed to explore these relationships. Finally, we did not have information on causes of death; thus, we could not relate the development of in-hospital sepsis with long-term mortality in any detail. In this regard, the development of sepsis after surgery may represent a causative factor or another marker of greater clinical fragility or both. Only interventional studies aimed at decreasing post-operative sepsis will provide a better understanding of the potential causative effect of post-operative sepsis on mortality.

## Conclusions

In a large epidemiological study of non-short-stay elective surgical patients, we found that approximately 1 in 65 patients developed post-operative sepsis and that, among survivors, 1 in 8 died within 1 year after discharge. The first month post-discharge was the highest-risk period for mortality, and older patients and those with co-morbidities were those most at risk. Strategies that target the first month after discharge in this group of patients should be considered and evaluated.

## Additional files

Additional file 1: Appendix 1. ICD-10-AM codes used to define postoperative sepsis. (DOCX 16 kb)
Additional file 2: Appendix 2. Codes used for exclusion criteria. (DOCX 104 kb)
Additional file 3: Appendix 3. Procedure codes from ICD-10-AM for selected surgical procedures. (DOCX 15 kb)
Additional file 4: Appendix 4. Results from sensitivity analyses. (DOCX 20 kb)

## Abbreviations

AAA: Abdominal aortic aneurysm
AHRQ: Agency for Healthcare Research and Quality
APDC: Admitted Patient Data Collection
CABG: Coronary artery bypass graft
ED: Emergency department
EVAR: Endovascular aneurysm repair
ICD-9-CM: International Classification of Diseases, Ninth Revision, Clinical Modification
ICD-10-AM: International Statistical Classification of Diseases and Related Health Problems, Tenth Revision, Australian Modification
ICU: Intensive care unit
IR: Incidence rate of sepsis reported per 1000 admissions
LOS: Length of stay
MDC: Major Diagnostic Categories
NNM: Nearest-neighbour matching
NSW: New South Wales
OECD: Organisation for Economic Co-operation and Development
PSM: Propensity score matching
RBDM: New South Wales Registry of Births, Deaths, and Marriages
SEIFA: Socio-Economic Indexes for Areas
