# Supplementary material for: The impact of post-operative sepsis on mortality after hospital discharge among elective surgical patients: a population-based cohort study
Source: Crit Care. 2017 Feb 20;21:34. doi: 10.1186/s13054-016-1596-7 (PMC5319141; doi:10.1186/s13054-016-1596-7)
Supplement: Additional file 1: Appendix 1. — ICD-10-AM codes used to define postoperative sepsis. (DOCX 16 kb) [file 13054_2016_1596_MOESM1_ESM.docx]

Appendix 1 ICD-10-AM codes used to define postoperative sepsis

| ICD 10 AM Code | Diagnosis |
| --- | --- |
| A40.0 | Sepsis due to streptococcus, group A |
| A40.1 | Sepsis due to streptococcus, group B |
| A40.2 | Sepsis due to streptococcus, group D |
| A40.3 | Sepsis due to Streptococcus pneumonia |
| A40.8 | Other streptococcal sepsis |
| A40.9 | Streptococcal sepsis, unspecified |
| A41.0 | Sepsis due to Staphylococcus aureus |
| A41.1 | Sepsis due to Coagulate-negative staphylococcus |
| A41.2 | Sepsis due to unspecified staphylococcus |
| A41.3 | Sepsis due to Haemophilus influenza |
| A41.4 | Sepsis due to anaerobes |
| A41.50 | Gram-negative septicaemia NOS |
| A41.51 | Sepsis due to Escherichia coli |
| A41.52 | Sepsis due to Pseudomonas |
| A41.58 | Sepsis due to other Gram-negative organisms |
| A41.8 | Other specified sepsis |
| A41.9 | Sepsis unspecified, septicaemia |
| R57.2 | Septic shock |
| R57.8 | Other shock |
| R65.0 | SIRS of infectious origin without acute organ failure |
| R65.1 | SIRS of infectious origin with acute organ failure |
| T81.1 | Shock during or resulting from a procedure, not elsewhere classified |

*SIRS=Systemic inflammatory response syndrome*
