# Supplementary material for: The impact of post-operative sepsis on mortality after hospital discharge among elective surgical patients: a population-based cohort study
Source: Crit Care. 2017 Feb 20;21:34. doi: 10.1186/s13054-016-1596-7 (PMC5319141; doi:10.1186/s13054-016-1596-7)
Supplement: Additional file 2: Appendix 2. — Codes used for exclusion criteria. (DOCX 104 kb) [file 13054_2016_1596_MOESM2_ESM.docx]

Appendix 2 Codes used for exclusion criteria

1. Cancer codes

| ICD–10 | Code description |
| --- | --- |
| C00.0 | Malignant neoplasm: External upper lip |
| C00.1 | Malignant neoplasm: External lower lip |
| C00.2 | Malignant neoplasm: External lip, unspecified |
| C00.3 | Malignant neoplasm: Upper lip, inner aspect |
| C00.4 | Malignant neoplasm: Lower lip, inner aspect |
| C00.5 | Malignant neoplasm: Lip, unspecified, inner aspect |
| C00.6 | Malignant neoplasm: Commissure of lip |
| C00.8 | Malignant neoplasm: Overlapping lesion of lip |
| C00.9 | Malignant neoplasm: Lip, unspecified |
| C01 | Malignant neoplasm of base of tongue |
| C02.0 | Malignant neoplasm: Dorsal surface of tongue |
| C02.1 | Malignant neoplasm: Border of tongue |
| C02.2 | Malignant neoplasm: Ventral surface of tongue |
| C02.3 | Malignant neoplasm: Anterior two-thirds of tongue, part unspecified |
| C02.4 | Malignant neoplasm: Lingual tonsil |
| C02.8 | Malignant neoplasm: Overlapping lesion of tongue |
| C02.9 | Malignant neoplasm: Tongue, unspecified |
| C03.0 | Malignant neoplasm: Upper gum |
| C03.1 | Malignant neoplasm: Lower gum |
| C03.9 | Malignant neoplasm: Gum, unspecified |
| C04.0 | Malignant neoplasm: Anterior floor of mouth |
| C04.1 | Malignant neoplasm: Lateral floor of mouth |
| C04.8 | Malignant neoplasm: Overlapping lesion of floor of mouth |
| C04.9 | Malignant neoplasm: Floor of mouth, unspecified |
| C05.0 | Malignant neoplasm: Hard palate |
| C05.1 | Malignant neoplasm: Soft palate |
| C05.2 | Malignant neoplasm: Uvula |
| C05.8 | Malignant neoplasm: Overlapping lesion of palate |
| C05.9 | Malignant neoplasm: Palate, unspecified |
| C06.0 | Malignant neoplasm: Cheek mucosa |
| C06.1 | Malignant neoplasm: Vestibule of mouth |
| C06.2 | Malignant neoplasm: Retromolar area |
| C06.8 | Malignant neoplasm: Overlapping lesion of other and unspecified parts of mouth |
| C06.9 | Malignant neoplasm: Mouth, unspecified |
| C07 | Malignant neoplasm of parotid gland |
| C08.0 | Malignant neoplasm: Submandibular gland |
| C08.1 | Malignant neoplasm: Sublingual gland |
| C08.8 | Malignant neoplasm: Overlapping lesion of major salivary glands |
| C08.9 | Malignant neoplasm: Major salivary gland, unspecified |
| C09.0 | Malignant neoplasm: Tonsillar fossa |
| C09.1 | Malignant neoplasm: Tonsillar pillar (anterior)(posterior) |
| C09.8 | Malignant neoplasm: Overlapping lesion of tonsil |
| C09.9 | Malignant neoplasm: Tonsil, unspecified |
| C10.0 | Malignant neoplasm: Vallecula |
| C10.1 | Malignant neoplasm: Anterior surface of epiglottis |
| C10.2 | Malignant neoplasm: Lateral wall of oropharynx |
| C10.3 | Malignant neoplasm: Posterior wall of oropharynx |
| C10.4 | Malignant neoplasm: Branchial cleft |
| C10.8 | Malignant neoplasm: Overlapping lesion of oropharynx |
| C10.9 | Malignant neoplasm: Oropharynx, unspecified |
| C11.0 | Malignant neoplasm: Superior wall of nasopharynx |
| C11.1 | Malignant neoplasm: Posterior wall of nasopharynx |
| C11.2 | Malignant neoplasm: Lateral wall of nasopharynx |
| C11.3 | Malignant neoplasm: Anterior wall of nasopharynx |
| C11.8 | Malignant neoplasm: Overlapping lesion of nasopharynx |
| C11.9 | Malignant neoplasm: Nasopharynx, unspecified |
| C12 | Malignant neoplasm of piriform sinus |
| C13.0 | Malignant neoplasm: Postcricoid region |
| C13.1 | Malignant neoplasm: Aryepiglottic fold, hypopharyngeal aspect |
| C13.2 | Malignant neoplasm: Posterior wall of hypopharynx |
| C13.8 | Malignant neoplasm: Overlapping lesion of hypopharynx |
| C13.9 | Malignant neoplasm: Hypopharynx, unspecified |
| C14.0 | Malignant neoplasm: Pharynx, unspecified |
| C14.2 | Malignant neoplasm: Waldeyer's ring |
| C14.8 | Malignant neoplasm: Overlapping lesion of lip, oral cavity and pharynx |
| C15.0 | Malignant neoplasm: Cervical part of oesophagus |
| C15.1 | Malignant neoplasm: Thoracic part of oesophagus |
| C15.2 | Malignant neoplasm: Abdominal part of oesophagus |
| C15.3 | Malignant neoplasm: Upper third of oesophagus |
| C15.4 | Malignant neoplasm: Middle third of oesophagus |
| C15.5 | Malignant neoplasm: Lower third of oesophagus |
| C15.8 | Malignant neoplasm: Overlapping lesion of oesophagus |
| C15.9 | Malignant neoplasm: Oesophagus, unspecified |
| C16.0 | Malignant neoplasm: Cardia |
| C16.1 | Malignant neoplasm: Fundus of stomach |
| C16.2 | Malignant neoplasm: Body of stomach |
| C16.3 | Malignant neoplasm: Pyloric antrum |
| C16.4 | Malignant neoplasm: Pylorus |
| C16.5 | Malignant neoplasm: Lesser curvature of stomach, unspecified |
| C16.6 | Malignant neoplasm: Greater curvature of stomach, unspecified |
| C16.8 | Malignant neoplasm: Overlapping lesion of stomach |
| C16.9 | Malignant neoplasm: Stomach, unspecified |
| C17.0 | Malignant neoplasm: Duodenum |
| C17.1 | Malignant neoplasm: Jejunum |
| C17.2 | Malignant neoplasm: Ileum |
| C17.3 | Malignant neoplasm: Meckel's diverticulum |
| C17.8 | Malignant neoplasm: Overlapping lesion of small intestine |
| C17.9 | Malignant neoplasm: Small intestine, unspecified |
| C18.0 | Malignant neoplasm: Caecum |
| C18.1 | Malignant neoplasm: Appendix |
| C18.2 | Malignant neoplasm: Ascending colon |
| C18.3 | Malignant neoplasm: Hepatic flexure |
| C18.4 | Malignant neoplasm: Transverse colon |
| C18.5 | Malignant neoplasm: Splenic flexure |
| C18.6 | Malignant neoplasm: Descending colon |
| C18.7 | Malignant neoplasm: Sigmoid colon |
| C18.8 | Malignant neoplasm: Overlapping lesion of colon |
| C18.9 | Malignant neoplasm: Colon, unspecified |
| C19 | Malignant neoplasm of rectosigmoid junction |
| C20 | Malignant neoplasm of rectum |
| C21.0 | Malignant neoplasm: Anus, unspecified |
| C21.1 | Malignant neoplasm: Anal canal |
| C21.2 | Malignant neoplasm: Cloacogenic zone |
| C21.8 | Malignant neoplasm: Overlapping lesion of rectum, anus and anal canal |
| C22.0 | Malignant neoplasm: Liver cell carcinoma |
| C22.1 | Malignant neoplasm: Intrahepatic bile duct carcinoma |
| C22.2 | Malignant neoplasm: Hepatoblastoma |
| C22.3 | Malignant neoplasm: Angiosarcoma of liver |
| C22.4 | Malignant neoplasm: Other sarcomas of liver |
| C22.7 | Malignant neoplasm: Other specified carcinomas of liver |
| C22.9 | Malignant neoplasm: Liver, unspecified |
| C23 | Malignant neoplasm of gallbladder |
| C24.0 | Malignant neoplasm: Extrahepatic bile duct |
| C24.1 | Malignant neoplasm: Ampulla of Vater |
| C24.8 | Malignant neoplasm: Overlapping lesion of biliary tract |
| C24.9 | Malignant neoplasm: Biliary tract, unspecified |
| C25.0 | Malignant neoplasm: Head of pancreas |
| C25.1 | Malignant neoplasm: Body of pancreas |
| C25.2 | Malignant neoplasm: Tail of pancreas |
| C25.3 | Malignant neoplasm: Pancreatic duct |
| C25.4 | Malignant neoplasm: Endocrine pancreas |
| C25.7 | Malignant neoplasm: Other parts of pancreas |
| C25.8 | Malignant neoplasm: Overlapping lesion of pancreas |
| C25.9 | Malignant neoplasm: Pancreas, unspecified |
| C26.0 | Malignant neoplasm: Intestinal tract, part unspecified |
| C26.1 | Malignant neoplasm: Spleen |
| C26.8 | Malignant neoplasm: Overlapping lesion of digestive system |
| C26.9 | Malignant neoplasm: Ill-defined sites within the digestive system |
| C30.0 | Malignant neoplasm: Nasal cavity |
| C30.1 | Malignant neoplasm: Middle ear |
| C31.0 | Malignant neoplasm: Maxillary sinus |
| C31.1 | Malignant neoplasm: Ethmoidal sinus |
| C31.2 | Malignant neoplasm: Frontal sinus |
| C31.3 | Malignant neoplasm: Sphenoidal sinus |
| C31.8 | Malignant neoplasm: Overlapping lesion of accessory sinuses |
| C31.9 | Malignant neoplasm: Accessory sinus, unspecified |
| C32.0 | Malignant neoplasm: Glottis |
| C32.1 | Malignant neoplasm: Supraglottis |
| C32.2 | Malignant neoplasm: Subglottis |
| C32.3 | Malignant neoplasm: Laryngeal cartilage |
| C32.8 | Malignant neoplasm: Overlapping lesion of larynx |
| C32.9 | Malignant neoplasm: Larynx, unspecified |
| C33 | Malignant neoplasm of trachea |
| C34.0 | Malignant neoplasm: Main bronchus |
| C34.1 | Malignant neoplasm: Upper lobe, bronchus or lung |
| C34.2 | Malignant neoplasm: Middle lobe, bronchus or lung |
| C34.3 | Malignant neoplasm: Lower lobe, bronchus or lung |
| C34.8 | Malignant neoplasm: Overlapping lesion of bronchus and lung |
| C34.9 | Malignant neoplasm: Bronchus or lung, unspecified |
| C37 | Malignant neoplasm of thymus |
| C38.0 | Malignant neoplasm: Heart |
| C38.1 | Malignant neoplasm: Anterior mediastinum |
| C38.2 | Malignant neoplasm: Posterior mediastinum |
| C38.3 | Malignant neoplasm: Mediastinum, part unspecified |
| C38.4 | Malignant neoplasm: Pleura |
| C38.8 | Malignant neoplasm: Overlapping lesion of heart, mediastinum and pleura |
| C39.0 | Malignant neoplasm: Upper respiratory tract, part unspecified |
| C39.8 | Malignant neoplasm: Overlapping lesion of respiratory and intrathoracic organs |
| C39.9 | Malignant neoplasm: Ill-defined sites within the respiratory system |
| C40.0 | Malignant neoplasm: Scapula and long bones of upper limb |
| C40.1 | Malignant neoplasm: Short bones of upper limb |
| C40.2 | Malignant neoplasm: Long bones of lower limb |
| C40.3 | Malignant neoplasm: Short bones of lower limb |
| C40.8 | Malignant neoplasm: Overlapping lesion of bone and articular cartilage of limbs |
| C40.9 | Malignant neoplasm: Bone and articular cartilage of limb, unspecified |
| C41.0 | Malignant neoplasm: Bones of skull and face |
| C41.1 | Malignant neoplasm: Mandible |
| C41.2 | Malignant neoplasm: Vertebral column |
| C41.3 | Malignant neoplasm: Ribs, sternum and clavicle |
| C41.4 | Malignant neoplasm: Pelvic bones, sacrum and coccyx |
| C41.8 | Malignant neoplasm: Overlapping lesion of bone and articular cartilage |
| C41.9 | Malignant neoplasm: Bone and articular cartilage, unspecified |
| C43.0 | Malignant neoplasm: Malignant melanoma of lip |
| C43.1 | Malignant neoplasm: Malignant melanoma of eyelid, including canthus |
| C43.2 | Malignant neoplasm: Malignant melanoma of ear and external auricular canal |
| C43.3 | Malignant neoplasm: Malignant melanoma of other and unspecified parts of face |
| C43.4 | Malignant neoplasm: Malignant melanoma of scalp and neck |
| C43.5 | Malignant neoplasm: Malignant melanoma of trunk |
| C43.6 | Malignant neoplasm: Malignant melanoma of upper limb, including shoulder |
| C43.7 | Malignant neoplasm: Malignant melanoma of lower limb, including hip |
| C43.8 | Malignant neoplasm: Overlapping malignant melanoma of skin |
| C43.9 | Malignant neoplasm: Malignant melanoma of skin, unspecified |
| C45.0 | Mesothelioma of pleura |
| C45.1 | Mesothelioma of peritoneum |
| C45.2 | Mesothelioma of pericardium |
| C45.7 | Mesothelioma of other sites |
| C45.9 | Mesothelioma, unspecified |
| C46.0 | Kaposi's sarcoma of skin |
| C46.1 | Kaposi's sarcoma of soft tissue |
| C46.2 | Kaposi's sarcoma of palate |
| C46.3 | Kaposi's sarcoma of lymph nodes |
| C46.7 | Kaposi's sarcoma of other sites |
| C46.8 | Kaposi's sarcoma of multiple organs |
| C46.9 | Kaposi's sarcoma, unspecified |
| C47.0 | Malignant neoplasm: Peripheral nerves of head, face and neck |
| C47.1 | Malignant neoplasm: Peripheral nerves of upper limb, including shoulder |
| C47.2 | Malignant neoplasm: Peripheral nerves of lower limb, including hip |
| C47.3 | Malignant neoplasm: Peripheral nerves of thorax |
| C47.4 | Malignant neoplasm: Peripheral nerves of abdomen |
| C47.5 | Malignant neoplasm: Peripheral nerves of pelvis |
| C47.6 | Malignant neoplasm: Peripheral nerves of trunk, unspecified |
| C47.8 | Malignant neoplasm: Overlapping lesion of peripheral nerves and autonomic nervous system |
| C47.9 | Malignant neoplasm: Peripheral nerves and autonomic nervous system, unspecified |
| C48.0 | Malignant neoplasm: Retroperitoneum |
| C48.1 | Malignant neoplasm: Specified parts of peritoneum |
| C48.2 | Malignant neoplasm: Peritoneum, unspecified |
| C48.8 | Malignant neoplasm: Overlapping lesion of retroperitoneum and peritoneum |
| C49.0 | Malignant neoplasm: Connective and soft tissue of head, face and neck |
| C49.1 | Malignant neoplasm: Connective and soft tissue of upper limb, including shoulder |
| C49.2 | Malignant neoplasm: Connective and soft tissue of lower limb, including hip |
| C49.3 | Malignant neoplasm: Connective and soft tissue of thorax |
| C49.4 | Malignant neoplasm: Connective and soft tissue of abdomen |
| C49.5 | Malignant neoplasm: Connective and soft tissue of pelvis |
| C49.6 | Malignant neoplasm: Connective and soft tissue of trunk, unspecified |
| C49.8 | Malignant neoplasm: Overlapping lesion of connective and soft tissue |
| C49.9 | Malignant neoplasm: Connective and soft tissue, unspecified |
| C50.0 | Malignant neoplasm: Nipple and areola |
| C50.1 | Malignant neoplasm: Central portion of breast |
| C50.2 | Malignant neoplasm: Upper-inner quadrant of breast |
| C50.3 | Malignant neoplasm: Lower-inner quadrant of breast |
| C50.4 | Malignant neoplasm: Upper-outer quadrant of breast |
| C50.5 | Malignant neoplasm: Lower-outer quadrant of breast |
| C50.6 | Malignant neoplasm: Axillary tail of breast |
| C50.8 | Malignant neoplasm: Overlapping lesion of breast |
| C50.9 | Malignant neoplasm: Breast, unspecified |
| C51.0 | Malignant neoplasm: Labium majus |
| C51.1 | Malignant neoplasm: Labium minus |
| C51.2 | Malignant neoplasm: Clitoris |
| C51.8 | Malignant neoplasm: Overlapping lesion of vulva |
| C51.9 | Malignant neoplasm: Vulva, unspecified |
| C52 | Malignant neoplasm of vagina |
| C53.0 | Malignant neoplasm: Endocervix |
| C53.1 | Malignant neoplasm: Exocervix |
| C53.8 | Malignant neoplasm: Overlapping lesion of cervix uteri |
| C53.9 | Malignant neoplasm: Cervix uteri, unspecified |
| C54.0 | Malignant neoplasm: Isthmus uteri |
| C54.1 | Malignant neoplasm: Endometrium |
| C54.2 | Malignant neoplasm: Myometrium |
| C54.3 | Malignant neoplasm: Fundus uteri |
| C54.8 | Malignant neoplasm: Overlapping lesion of corpus uteri |
| C54.9 | Malignant neoplasm: Corpus uteri, unspecified |
| C55 | Malignant neoplasm of uterus, part unspecified |
| C56 | Malignant neoplasm of ovary |
| C57.0 | Malignant neoplasm: Fallopian tube |
| C57.1 | Malignant neoplasm: Broad ligament |
| C57.2 | Malignant neoplasm: Round ligament |
| C57.3 | Malignant neoplasm: Parametrium |
| C57.4 | Malignant neoplasm: Uterine adnexa, unspecified |
| C57.7 | Malignant neoplasm: Other specified female genital organs |
| C57.8 | Malignant neoplasm: Overlapping lesion of female genital organs |
| C57.9 | Malignant neoplasm: Female genital organ, unspecified |
| C58 | Malignant neoplasm of placenta |
| C60.0 | Malignant neoplasm: Prepuce |
| C60.1 | Malignant neoplasm: Glans penis |
| C60.2 | Malignant neoplasm: Body of penis |
| C60.8 | Malignant neoplasm: Overlapping lesion of penis |
| C60.9 | Malignant neoplasm: Penis, unspecified |
| C61 | Malignant neoplasm of prostate |
| C62.0 | Malignant neoplasm: Undescended testis |
| C62.1 | Malignant neoplasm: Descended testis |
| C62.9 | Malignant neoplasm: Testis, unspecified |
| C63.0 | Malignant neoplasm: Epididymis |
| C63.1 | Malignant neoplasm: Spermatic cord |
| C63.2 | Malignant neoplasm: Scrotum |
| C63.7 | Malignant neoplasm: Other specified male genital organs |
| C63.8 | Malignant neoplasm: Overlapping lesion of male genital organs |
| C63.9 | Malignant neoplasm: Male genital organ, unspecified |
| C64 | Malignant neoplasm of kidney, except renal pelvis |
| C65 | Malignant neoplasm of renal pelvis |
| C66 | Malignant neoplasm of ureter |
| C67.0 | Malignant neoplasm: Trigone of bladder |
| C67.1 | Malignant neoplasm: Dome of bladder |
| C67.2 | Malignant neoplasm: Lateral wall of bladder |
| C67.3 | Malignant neoplasm: Anterior wall of bladder |
| C67.4 | Malignant neoplasm: Posterior wall of bladder |
| C67.5 | Malignant neoplasm: Bladder neck |
| C67.6 | Malignant neoplasm: Ureteric orifice |
| C67.7 | Malignant neoplasm: Urachus |
| C67.8 | Malignant neoplasm: Overlapping lesion of bladder |
| C67.9 | Malignant neoplasm: Bladder, unspecified |
| C68.0 | Malignant neoplasm: Urethra |
| C68.1 | Malignant neoplasm: Paraurethral gland |
| C68.8 | Malignant neoplasm: Overlapping lesion of urinary organs |
| C68.9 | Malignant neoplasm: Urinary organ, unspecified |
| C69.0 | Malignant neoplasm: Conjunctiva |
| C69.1 | Malignant neoplasm: Cornea |
| C69.2 | Malignant neoplasm: Retina |
| C69.3 | Malignant neoplasm: Choroid |
| C69.4 | Malignant neoplasm: Ciliary body |
| C69.5 | Malignant neoplasm: Lacrimal gland and duct |
| C69.6 | Malignant neoplasm: Orbit |
| C69.8 | Malignant neoplasm: Overlapping lesion of eye and adnexa |
| C69.9 | Malignant neoplasm: Eye, unspecified |
| C70.0 | Malignant neoplasm: Cerebral meninges |
| C70.1 | Malignant neoplasm: Spinal meninges |
| C70.9 | Malignant neoplasm: Meninges, unspecified |
| C71.0 | Malignant neoplasm: Cerebrum, except lobes and ventricles |
| C71.1 | Malignant neoplasm: Frontal lobe |
| C71.2 | Malignant neoplasm: Temporal lobe |
| C71.3 | Malignant neoplasm: Parietal lobe |
| C71.4 | Malignant neoplasm: Occipital lobe |
| C71.5 | Malignant neoplasm: Cerebral ventricle |
| C71.6 | Malignant neoplasm: Cerebellum |
| C71.7 | Malignant neoplasm: Brain stem |
| C71.8 | Malignant neoplasm: Overlapping lesion of brain |
| C71.9 | Malignant neoplasm: Brain, unspecified |
| C72.0 | Malignant neoplasm: Spinal cord |
| C72.1 | Malignant neoplasm: Cauda equina |
| C72.2 | Malignant neoplasm: Olfactory nerve |
| C72.3 | Malignant neoplasm: Optic nerve |
| C72.4 | Malignant neoplasm: Acoustic nerve |
| C72.5 | Malignant neoplasm: Other and unspecified cranial nerves |
| C72.8 | Malignant neoplasm: Overlapping lesion of brain and other parts of central nervous system |
| C72.9 | Malignant neoplasm: Central nervous system, unspecified |
| C73 | Malignant neoplasm of thyroid gland |
| C74.0 | Malignant neoplasm: Cortex of adrenal gland |
| C74.1 | Malignant neoplasm: Medulla of adrenal gland |
| C74.9 | Malignant neoplasm: Adrenal gland, unspecified |
| C75.0 | Malignant neoplasm: Parathyroid gland |
| C75.1 | Malignant neoplasm: Pituitary gland |
| C75.2 | Malignant neoplasm: Craniopharyngeal duct |
| C75.3 | Malignant neoplasm: Pineal gland |
| C75.4 | Malignant neoplasm: Carotid body |
| C75.5 | Malignant neoplasm: Aortic body and other paraganglia |
| C75.8 | Malignant neoplasm: Pluriglandular involvement, unspecified |
| C75.9 | Malignant neoplasm: Endocrine gland, unspecified |
| C76.0 | Malignant neoplasm of other and ill-defined sites: Head, face and neck |
| C76.1 | Malignant neoplasm of other and ill-defined sites: Thorax |
| C76.2 | Malignant neoplasm of other and ill-defined sites: Abdomen |
| C76.3 | Malignant neoplasm of other and ill-defined sites: Pelvis |
| C76.4 | Malignant neoplasm of other and ill-defined sites: Upper limb |
| C76.5 | Malignant neoplasm of other and ill-defined sites: Lower limb |
| C76.7 | Malignant neoplasm of other and ill-defined sites: Other ill-defined sites |
| C76.8 | Malignant neoplasm of other and ill-defined sites: Overlapping lesion of other and ill-defined sites |
| C77.0 | Secondary and unspecified malignant neoplasm: Lymph nodes of head, face and neck |
| C77.1 | Secondary and unspecified malignant neoplasm: Intrathoracic lymph nodes |
| C77.2 | Secondary and unspecified malignant neoplasm: Intra-abdominal lymph nodes |
| C77.3 | Secondary and unspecified malignant neoplasm: Axillary and upper limb lymph nodes |
| C77.4 | Secondary and unspecified malignant neoplasm: Inguinal and lower limb lymph nodes |
| C77.5 | Secondary and unspecified malignant neoplasm: Intrapelvic lymph nodes |
| C77.8 | Secondary and unspecified malignant neoplasm: Lymph nodes of multiple regions |
| C77.9 | Secondary and unspecified malignant neoplasm: Lymph node, unspecified |
| C78.0 | Secondary malignant neoplasm of lung |
| C78.1 | Secondary malignant neoplasm of mediastinum |
| C78.2 | Secondary malignant neoplasm of pleura |
| C78.3 | Secondary malignant neoplasm of other and unspecified respiratory organs |
| C78.4 | Secondary malignant neoplasm of small intestine |
| C78.5 | Secondary malignant neoplasm of large intestine and rectum |
| C78.6 | Secondary malignant neoplasm of retroperitoneum and peritoneum |
| C78.7 | Secondary malignant neoplasm of liver |
| C78.8 | Secondary malignant neoplasm of other and unspecified digestive organs |
| C79.0 | Secondary malignant neoplasm of kidney and renal pelvis |
| C79.1 | Secondary malignant neoplasm of bladder and other and unspecified urinary organs |
| C79.2 | Secondary malignant neoplasm of skin |
| C79.3 | Secondary malignant neoplasm of brain and cerebral meninges |
| C79.4 | Secondary malignant neoplasm of other and unspecified parts of nervous system |
| C79.5 | Secondary malignant neoplasm of bone and bone marrow |
| C79.6 | Secondary malignant neoplasm of ovary |
| C79.7 | Secondary malignant neoplasm of adrenal gland |
| C79.8 | Secondary malignant neoplasm of other specified sites |
| C80 | Malignant neoplasm without specification of site |
| C81.0 | Hodgkin's disease: Lymphocytic predominance |
| C81.1 | Hodgkin's disease: Nodular sclerosis |
| C81.2 | Hodgkin's disease: Mixed cellularity |
| C81.3 | Hodgkin's disease: Lymphocytic depletion |
| C81.7 | Hodgkin's disease: Other Hodgkin's disease |
| C81.9 | Hodgkin's disease: Hodgkin's disease, unspecified |
| C82.0 | Non-Hodgkin's lymphoma: Small cleaved cell, follicular |
| C82.1 | Non-Hodgkin's lymphoma: Mixed small cleaved and large cell, follicular |
| C82.2 | Non-Hodgkin's lymphoma: Large cell, follicular |
| C82.7 | Other types of follicular non-Hodgkin's lymphoma |
| C82.9 | Follicular non-Hodgkin's lymphoma, unspecified |
| C83.0 | Non-Hodgkin's lymphoma: Small cell (diffuse) |
| C83.1 | Non-Hodgkin's lymphoma: Small cleaved cell (diffuse) |
| C83.2 | Non-Hodgkin's lymphoma: Mixed small and large cell (diffuse) |
| C83.3 | Non-Hodgkin's lymphoma: Large cell (diffuse) |
| C83.4 | Non-Hodgkin's lymphoma: Immunoblastic (diffuse) |
| C83.5 | Non-Hodgkin's lymphoma: Lymphoblastic (diffuse) |
| C83.6 | Non-Hodgkin's lymphoma: Undifferentiated (diffuse) |
| C83.7 | Burkitt's tumour |
| C83.8 | Other types of diffuse non-Hodgkin's lymphoma |
| C83.9 | Diffuse non-Hodgkin's lymphoma, unspecified |
| C84.0 | Mycosis fungoides |
| C84.1 | Sezary's disease |
| C84.2 | T-zone lymphoma |
| C84.3 | Lymphoepithelioid lymphoma |
| C84.4 | Peripheral T-cell lymphoma |
| C84.5 | Other and unspecified T-cell lymphomas |
| C85.0 | Lymphosarcoma |
| C85.1 | B-cell lymphoma, unspecified |
| C85.7 | Other specified types of non-Hodgkin's lymphoma |
| C85.9 | Non-Hodgkin's lymphoma, unspecified type |
| C88.0 | Waldenstr¸m's macroglobulinaemia |
| C90.0 | Multiple myeloma |
| C90.1 | Plasma cell leukaemia |
| C90.2 | Plasmacytoma, extramedullary |
| C91.0 | Acute lymphoblastic leukaemia |
| C91.1 | Chronic lymphocytic leukaemia |
| C91.2 | Subacute lymphocytic leukaemia |
| C91.3 | Prolymphocytic leukaemia |
| C91.4 | Hairy-cell leukaemia |
| C91.5 | Adult T-cell leukaemia |
| C91.7 | Other lymphoid leukaemia |
| C91.9 | Lymphoid leukaemia, unspecified |
| C92.0 | Acute myeloid leukaemia |
| C92.1 | Chronic myeloid leukaemia |
| C92.2 | Subacute myeloid leukaemia |
| C92.3 | Myeloid sarcoma |
| C92.4 | Acute promyelocytic leukaemia |
| C92.5 | Acute myelomonocytic leukaemia |
| C92.7 | Other myeloid leukaemia |
| C92.9 | Myeloid leukaemia, unspecified |
| C93.0 | Acute monocytic leukaemia |
| C93.1 | Chronic monocytic leukaemia |
| C93.2 | Subacute monocytic leukaemia |
| C93.7 | Other monocytic leukaemia |
| C93.9 | Monocytic leukaemia, unspecified |
| C94.0 | Acute erythraemia and erythroleukaemia |
| C94.1 | Chronic erythraemia |
| C94.2 | Acute megakaryoblastic leukaemia |
| C94.3 | Mast cell leukaemia |
| C94.4 | Acute panmyelosis |
| C94.5 | Acute myelofibrosis |
| C94.7 | Other specified leukaemias |
| C95.0 | Acute leukaemia of unspecified cell type |
| C95.1 | Chronic leukaemia of unspecified cell type |
| C95.2 | Subacute leukaemia of unspecified cell type |
| C95.7 | Other leukaemia of unspecified cell type |
| C95.9 | Leukaemia, unspecified |
| C96.0 | Letterer-Siwe disease |
| C96.1 | Malignant histiocytosis |
| C96.2 | Malignant mast cell tumour |
| C96.3 | True histiocytic lymphoma |
| C96.7 | Other specified malignant neoplasms of lymphoid, haematopoietic and related tissue |
| C96.9 | Malignant neoplasm of lymphoid, haematopoietic and related tissue, unspecified |
| C97 | Malignant neoplasms of independent (primary) multiple sites |
| Z85.0 | Personal history of malignant neoplasm of digestive organs |
| Z85.1 | Personal history of malignant neoplasm of trachea, bronchus and lung |
| Z85.2 | Personal history of malignant neoplasm of other respiratory and intrathoracic organs |
| Z85.3 | Personal history of malignant neoplasm of breast |
| Z85.4 | Personal history of malignant neoplasm of genital organs |
| Z85.5 | Personal history of malignant neoplasm of urinary tract |
| Z85.6 | Personal history of leukaemia |
| Z85.7 | Personal history of other malignant neoplasms of lymphoid, haematopoietic and related tissues |
| Z85.8 | Personal history of malignant neoplasms of other organs and systems |
| Z85.9 | Personal history of malignant neoplasm, unspecified |

1. Infection codes

| ICD–10 | Code description |
| --- | --- |
| A00.0 | Cholera due to Vibrio cholerae 01, biovar cholerae |
| A00.1 | Cholera due to Vibrio cholerae 01, biovar eltor |
| A00.9 | Cholera, unspecified |
| A01.0 | Typhoid fever |
| A01.1 | Paratyphoid fever A |
| A01.2 | Paratyphoid fever B |
| A01.3 | Paratyphoid fever C |
| A01.4 | Paratyphoid fever, unspecified |
| A02.0 | Salmonella enteritis |
| A02.1 | Salmonella septicaemia |
| A02.2 | Localized salmonella infections |
| A02.8 | Other specified salmonella infections |
| A02.9 | Salmonella infection, unspecified |
| A03.0 | Shigellosis due to Shigella dysenteriae |
| A03.1 | Shigellosis due to Shigella flexneri |
| A03.2 | Shigellosis due to Shigella boydii |
| A03.3 | Shigellosis due to Shigella sonnei |
| A03.8 | Other shigellosis |
| A03.9 | Shigellosis, unspecified |
| A04.0 | Enteropathogenic Escherichia coli infection |
| A04.1 | Enterotoxigenic Escherichia coli infection |
| A04.2 | Enteroinvasive Escherichia coli infection |
| A04.3 | Enterohaemorrhagic Escherichia coli infection |
| A04.4 | Other intestinal Escherichia coli infections |
| A04.5 | Campylobacter enteritis |
| A04.6 | Enteritis due to Yersinia enterocolitica |
| A04.7 | Enterocolitis due to Clostridium difficile |
| A04.8 | Other specified bacterial intestinal infections |
| A04.9 | Bacterial intestinal infection, unspecified |
| A05.0 | Foodborne staphylococcal intoxication |
| A05.1 | Botulism |
| A05.2 | Foodborne Clostridium perfringens [Clostridium welchii] intoxication |
| A05.3 | Foodborne Vibrio parahaemolyticus intoxication |
| A05.4 | Foodborne Bacillus cereus intoxication |
| A05.8 | Other specified bacterial foodborne intoxications |
| A05.9 | Bacterial foodborne intoxication, unspecified |
| A20.0 | Bubonic plague |
| A20.1 | Cellulocutaneous plague |
| A20.2 | Pneumonic plague |
| A20.3 | Plague meningitis |
| A20.7 | Septicaemic plague |
| A20.8 | Other forms of plague |
| A20.9 | Plague, unspecified |
| A21.0 | Ulceroglandular tularaemia |
| A21.1 | Oculoglandular tularaemia |
| A21.2 | Pulmonary tularaemia |
| A21.3 | Gastrointestinal tularaemia |
| A21.7 | Generalized tularaemia |
| A21.8 | Other forms of tularaemia |
| A21.9 | Tularaemia, unspecified |
| A22.0 | Cutaneous anthrax |
| A22.1 | Pulmonary anthrax |
| A22.2 | Gastrointestinal anthrax |
| A22.7 | Anthrax septicaemia |
| A22.8 | Other forms of anthrax |
| A22.9 | Anthrax, unspecified |
| A23.0 | Brucellosis due to Brucella melitensis |
| A23.1 | Brucellosis due to Brucella abortus |
| A23.2 | Brucellosis due to Brucella suis |
| A23.3 | Brucellosis due to Brucella canis |
| A23.8 | Other brucellosis |
| A23.9 | Brucellosis, unspecified |
| A24.0 | Glanders |
| A24.1 | Acute and fulminating melioidosis |
| A24.2 | Subacute and chronic melioidosis |
| A24.3 | Other melioidosis |
| A24.4 | Melioidosis, unspecified |
| A25.0 | Spirillosis |
| A25.1 | Streptobacillosis |
| A25.9 | Rat-bite fever, unspecified |
| A26.0 | Cutaneous erysipeloid |
| A26.7 | Erysipelothrix septicaemia |
| A26.8 | Other forms of erysipeloid |
| A26.9 | Erysipeloid, unspecified |
| A28.0 | Pasteurellosis |
| A28.1 | Cat-scratch disease |
| A28.2 | Extraintestinal yersiniosis |
| A28.8 | Other specified zoonotic bacterial diseases, not elsewhere classified |
| A28.9 | Zoonotic bacterial disease, unspecified |
| A32.0 | Cutaneous listeriosis |
| A32.1 | Listerial meningitis and meningoencephalitis |
| A32.7 | Listerial septicaemia |
| A32.8 | Other forms of listeriosis |
| A32.9 | Listeriosis, unspecified |
| A33 | Tetanus neonatorum |
| A34 | Obstetrical tetanus |
| A35 | Other tetanus |
| A36.0 | Pharyngeal diphtheria |
| A36.1 | Nasopharyngeal diphtheria |
| A36.2 | Laryngeal diphtheria |
| A36.3 | Cutaneous diphtheria |
| A36.8 | Other diphtheria |
| A36.9 | Diphtheria, unspecified |
| A37.0 | Whooping cough due to Bordetella pertussis |
| A37.1 | Whooping cough due to Bordetella parapertussis |
| A37.8 | Whooping cough due to other Bordetella species |
| A37.9 | Whooping cough, unspecified |
| A38 | Scarlet fever |
| A39.0 | Meningococcal meningitis |
| A39.1 | Waterhouse-Friderichsen syndrome |
| A39.2 | Acute meningococcaemia |
| A39.3 | Chronic meningococcaemia |
| A39.4 | Meningococcaemia, unspecified |
| A39.5 | Meningococcal heart disease |
| A39.8 | Other meningococcal infections |
| A39.9 | Meningococcal infection, unspecified |
| A40.0 | Septicaemia due to streptococcus, group A |
| A40.1 | Septicaemia due to streptococcus, group B |
| A40.2 | Septicaemia due to streptococcus, group D |
| A40.3 | Septicaemia due to Streptococcus pneumoniae |
| A40.8 | Other streptococcal septicaemia |
| A40.9 | Streptococcal septicaemia, unspecified |
| A41.0 | Septicaemia due to Staphylococcus aureus |
| A41.1 | Septicaemia due to other specified staphylococcus |
| A41.2 | Septicaemia due to unspecified staphylococcus |
| A41.3 | Septicaemia due to Haemophilus influenzae |
| A41.4 | Septicaemia due to anaerobes |
| A41.5 | Septicaemia due to other Gram-negative organisms |
| A41.8 | Other specified septicaemia |
| A41.9 | Septicaemia, unspecified |
| A42.0 | Pulmonary actinomycosis |
| A42.1 | Abdominal actinomycosis |
| A42.2 | Cervicofacial actinomycosis |
| A42.7 | Actinomycotic septicaemia |
| A42.8 | Other forms of actinomycosis |
| A42.9 | Actinomycosis, unspecified |
| A43.0 | Pulmonary nocardiosis |
| A43.1 | Cutaneous nocardiosis |
| A43.8 | Other forms of nocardiosis |
| A43.9 | Nocardiosis, unspecified |
| A46 | Erysipelas |
| A48.0 | Gas gangrene |
| A48.1 | Legionnaires' disease |
| A48.2 | Nonpneumonic Legionnaires' disease [Pontiac fever] |
| A48.3 | Toxic shock syndrome |
| A48.4 | Brazilian purpuric fever |
| A48.8 | Other specified bacterial diseases |
| A49.0 | Staphylococcal infection, unspecified |
| A49.1 | Streptococcal infection, unspecified |
| A49.2 | Haemophilus influenzae infection, unspecified |
| A49.3 | Mycoplasma infection, unspecified |
| A49.8 | Other bacterial infections of unspecified site |
| A49.9 | Bacterial infection, unspecified |
| A54.0 | Gonococcal infection of lower genitourinary tract without periurethral or accessory gland abscess |
| A54.1 | Gonococcal infection of lower genitourinary tract with periurethral and accessory gland abscess |
| A54.2 | Gonococcal pelviperitonitis and other gonococcal genitourinary infections |
| A54.3 | Gonococcal infection of eye |
| A54.4 | Gonococcal infection of musculoskeletal system |
| A54.5 | Gonococcal pharyngitis |
| A54.6 | Gonococcal infection of anus and rectum |
| A54.8 | Other gonococcal infections |
| A54.9 | Gonococcal infection, unspecified |
| B95.0 | Streptococcus, group A, as the cause of diseases classified to other chapters |
| B95.1 | Streptococcus, group B, as the cause of diseases classified to other chapters |
| B95.2 | Streptococcus, group D, as the cause of diseases classified to other chapters |
| B95.3 | Streptococcus pneumoniae as the cause of diseases classified to other chapters |
| B95.4 | Other streptococcus as the cause of diseases classified to other chapters |
| B95.5 | Unspecified streptococcus as the cause of diseases classified to other chapters |
| B95.6 | Staphylococcus aureus as the cause of diseases classified to other chapters |
| B95.7 | Other staphylococcus as the cause of diseases classified to other chapters |
| B95.8 | Unspecified staphylococcus as the cause of diseases classified to other chapters |
| B96.0 | Mycoplasma pneumoniae [M. pneumoniae] as the cause of diseases classified to other chapters |
| B96.1 | Klebsiella pneumoniae [K. pneumoniae] as the cause of diseases classified to other chapters |
| B96.2 | Escherichia coli [E. coli] as the cause of diseases classified to other chapters |
| B96.3 | Haemophilus influenzae [H. influenzae] as the cause of diseases classified to other chapters |
| B96.4 | Proteus (mirabilis)(morganii) as the cause of diseases classified to other chapters |
| B96.5 | Pseudomonas (aeruginosa)(mallei)(pseudomallei) as the cause of diseases classified to other chapters |
| B96.6 | Bacillus fragilis [B. fragilis] as the cause of diseases classified to other chapters |
| B96.7 | Clostridium perfringens [C. perfringens] as the cause of diseases classified to other chapters |
| B96.8 | Other specified bacterial agents as the cause of diseases classified to other chapters |
| D73.3 | Abscess of spleen |
| E32.1 | Abscess of thymus |
| G00.0 | Haemophilus meningitis |
| G00.1 | Pneumococcal meningitis |
| G00.2 | Streptococcal meningitis |
| G00.3 | Staphylococcal meningitis |
| G00.8 | Other bacterial meningitis |
| G00.9 | Bacterial meningitis, unspecified |
| G03.8 | Meningitis due to other specified causes |
| G03.9 | Meningitis, unspecified |
| G04.2 | Bacterial meningoencephalitis and meningomyelitis, not elsewhere classified |
| G06.0 | Intracranial abscess and granuloma |
| G06.1 | Intraspinal abscess and granuloma |
| G06.2 | Extradural and subdural abscess, unspecified |
| G07 | Intracranial and intraspinal abscess and granuloma in diseases classified elsewhere |
| H00.0 | Hordeolum and other deep inflammation of eyelid |
| H01.0 | Blepharitis |
| H01.8 | Other specified inflammation of eyelid |
| H04.0 | Dacryoadenitis |
| H04.3 | Acute and unspecified inflammation of lacrimal passages |
| H04.4 | Chronic inflammation of lacrimal passages |
| H05.0 | Acute inflammation of orbit |
| H10.0 | Mucopurulent conjunctivitis |
| H10.2 | Other acute conjunctivitis |
| H10.3 | Acute conjunctivitis, unspecified |
| H10.5 | Blepharoconjunctivitis |
| H10.8 | Other conjunctivitis |
| H10.9 | Conjunctivitis, unspecified |
| H13.1 | Conjunctivitis in infectious and parasitic diseases classified elsewhere |
| H44.0 | Purulent endophthalmitis |
| H60.0 | Abscess of external ear |
| H60.1 | Cellulitis of external ear |
| H60.2 | Malignant otitis externa |
| H60.3 | Other infective otitis externa |
| H60.9 | Otitis externa, unspecified |
| H66.0 | Acute suppurative otitis media |
| H66.1 | Chronic tubotympanic suppurative otitis media |
| H66.2 | Chronic atticoantral suppurative otitis media |
| H66.3 | Other chronic suppurative otitis media |
| H66.4 | Suppurative otitis media, unspecified |
| H66.9 | Otitis media, unspecified |
| H68.0 | Eustachian salpingitis |
| H70.0 | Acute mastoiditis |
| H70.2 | Petrositis |
| H73.0 | Acute myringitis |
| H83.0 | Labyrinthitis |
| I30.1 | Infective pericarditis |
| I30.8 | Other forms of acute pericarditis |
| I30.9 | Acute pericarditis, unspecified |
| I32.0 | Pericarditis in bacterial diseases classified elsewhere |
| I33.0 | Acute and subacute infective endocarditis |
| I33.9 | Acute endocarditis, unspecified |
| I40.0 | Infective myocarditis |
| J01.0 | Acute maxillary sinusitis |
| J01.1 | Acute frontal sinusitis |
| J01.2 | Acute ethmoidal sinusitis |
| J01.3 | Acute sphenoidal sinusitis |
| J01.4 | Acute pansinusitis |
| J01.8 | Other acute sinusitis |
| J01.9 | Acute sinusitis, unspecified |
| J02.0 | Streptococcal pharyngitis |
| J02.8 | Acute pharyngitis due to other specified organisms |
| J02.9 | Acute pharyngitis, unspecified |
| J03.0 | Streptococcal tonsillitis |
| J03.8 | Acute tonsillitis due to other specified organisms |
| J03.9 | Acute tonsillitis, unspecified |
| J05.1 | Acute epiglottitis |
| J13 | Pneumonia due to Streptococcus pneumoniae |
| J14 | Pneumonia due to Haemophilus influenzae |
| J15.0 | Pneumonia due to Klebsiella pneumoniae |
| J15.1 | Pneumonia due to Pseudomonas |
| J15.2 | Pneumonia due to staphylococcus |
| J15.3 | Pneumonia due to streptococcus, group B |
| J15.4 | Pneumonia due to other streptococci |
| J15.5 | Pneumonia due to Escherichia coli |
| J15.6 | Pneumonia due to other aerobic Gram-negative bacteria |
| J15.7 | Pneumonia due to Mycoplasma pneumoniae |
| J15.8 | Other bacterial pneumonia |
| J15.9 | Bacterial pneumonia, unspecified |
| J17.0 | Pneumonia in bacterial diseases classified elsewhere |
| J18.0 | Bronchopneumonia, unspecified |
| J18.1 | Lobar pneumonia, unspecified |
| J18.8 | Other pneumonia, organism unspecified |
| J20.0 | Acute bronchitis due to Mycoplasma pneumoniae |
| J20.1 | Acute bronchitis due to Haemophilus influenzae |
| J20.2 | Acute bronchitis due to streptococcus |
| J34.0 | Abscess, furuncle and carbuncle of nose |
| J36 | Peritonsillar abscess |
| J39.0 | Retropharyngeal and parapharyngeal abscess |
| J39.1 | Other abscess of pharynx |
| J40 | Bronchitis, not specified as acute or chronic |
| J44.0 | Chronic obstructive pulmonary disease with acute lower respiratory infection |
| J85.0 | Gangrene and necrosis of lung |
| J85.1 | Abscess of lung with pneumonia |
| J85.2 | Abscess of lung without pneumonia |
| J85.3 | Abscess of mediastinum |
| J86.0 | Pyothorax with fistula |
| J86.9 | Pyothorax without fistula |
| J90 | Pleural effusion, not elsewhere classified |
| J98.5 | Diseases of mediastinum, not elsewhere classified |
| K04.0 | Pulpitis |
| K04.6 | Periapical abscess with sinus |
| K04.7 | Periapical abscess without sinus |
| K05.0 | Acute gingivitis |
| K05.2 | Acute periodontitis |
| K10.2 | Inflammatory conditions of jaws |
| K11.3 | Abscess of salivary gland |
| K12.2 | Cellulitis and abscess of mouth |
| K35.0 | Acute appendicitis with generalized peritonitis |
| K35.1 | Acute appendicitis with peritoneal abscess |
| K35.9 | Acute appendicitis, unspecified |
| K36 | Other appendicitis |
| K37 | Unspecified appendicitis |
| K57.0 | Diverticular disease of small intestine with perforation and abscess |
| K57.1 | Diverticular disease of small intestine without perforation or abscess |
| K57.2 | Diverticular disease of large intestine with perforation and abscess |
| K57.3 | Diverticular disease of large intestine without perforation or abscess |
| K57.4 | Diverticular disease of both small and large intestine with perforation and abscess |
| K57.5 | Diverticular disease of both small and large intestine without perforation or abscess |
| K57.8 | Diverticular disease of intestine, part unspecified, with perforation and abscess |
| K57.9 | Diverticular disease of intestine, part unspecified, without perforation or abscess |
| K61.0 | Anal abscess |
| K61.1 | Rectal abscess |
| K61.2 | Anorectal abscess |
| K61.3 | Ischiorectal abscess |
| K61.4 | Intrasphincteric abscess |
| K63.0 | Abscess of intestine |
| K65.0 | Acute peritonitis |
| K65.8 | Other peritonitis |
| K65.9 | Peritonitis, unspecified |
| K67.1 | Gonococcal peritonitis |
| K75.0 | Abscess of liver |
| K75.1 | Phlebitis of portal vein |
| K80.0 | Calculus of gallbladder with acute cholecystitis |
| K80.3 | Calculus of bile duct with cholangitis |
| K80.4 | Calculus of bile duct with cholecystitis |
| K81.0 | Acute cholecystitis |
| K81.8 | Other cholecystitis |
| K81.9 | Cholecystitis, unspecified |
| K82.2 | Perforation of gallbladder |
| K83.0 | Cholangitis |
| K83.2 | Perforation of bile duct |
| K85.0 | Idiopathic acute pancreatitis |
| K85.1 | Biliary acute pancreatitis |
| K85.2 | Alcohol-induced acute pancreatitis |
| K85.3 | Drug-induced acute pancreatitis |
| K85.8 | Other acute pancreatitis |
| K85.9 | Acute pancreatitis, unspecified |
| L00 | Staphylococcal scalded skin syndrome |
| L01.0 | Impetigo [any organism] [any site] |
| L01.1 | Impetiginization of other dermatoses |
| L02.0 | Cutaneous abscess, furuncle and carbuncle of face |
| L02.1 | Cutaneous abscess, furuncle and carbuncle of neck |
| L02.2 | Cutaneous abscess, furuncle and carbuncle of trunk |
| L02.3 | Cutaneous abscess, furuncle and carbuncle of buttock |
| L02.4 | Cutaneous abscess, furuncle and carbuncle of limb |
| L02.8 | Cutaneous abscess, furuncle and carbuncle of other sites |
| L02.9 | Cutaneous abscess, furuncle and carbuncle, unspecified |
| L03.0 | Cellulitis of finger and toe |
| L03.1 | Cellulitis of other parts of limb |
| L03.2 | Cellulitis of face |
| L03.3 | Cellulitis of trunk |
| L03.8 | Cellulitis of other sites |
| L03.9 | Cellulitis, unspecified |
| L04.0 | Acute lymphadenitis of face, head and neck |
| L04.1 | Acute lymphadenitis of trunk |
| L04.2 | Acute lymphadenitis of upper limb |
| L04.3 | Acute lymphadenitis of lower limb |
| L04.8 | Acute lymphadenitis of other sites |
| L04.9 | Acute lymphadenitis, unspecified |
| L05.0 | Pilonidal cyst with abscess |
| L08.0 | Pyoderma |
| L08.8 | Other specified local infections of skin and subcutaneous tissue |
| L08.9 | Local infection of skin and subcutaneous tissue, unspecified |
| L30.3 | Infective dermatitis |
| M00.0 | Staphylococcal arthritis and polyarthritis |
| M00.1 | Pneumococcal arthritis and polyarthritis |
| M00.2 | Other streptococcal arthritis and polyarthritis |
| M00.8 | Arthritis and polyarthritis due to other specified bacterial agents |
| M00.9 | Pyogenic arthritis, unspecified |
| M03.0 | Postmeningococcal arthritis |
| M03.1 | Postinfective arthropathy in syphilis |
| M03.2 | Other postinfectious arthropathies in diseases classified elsewhere |
| M03.6 | Reactive arthropathy in other diseases classified elsewhere |
| M46.2 | Osteomyelitis of vertebra |
| M60.0 | Infective myositis |
| M63.0 | Myositis in bacterial diseases classified elsewhere |
| M72..6 | Necrotizing fasciitis |
| M73.0 | Gonococcal bursitis |
| M86.0 | Acute haematogenous osteomyelitis |
| M86.1 | Other acute osteomyelitis |
| M86.2 | Subacute osteomyelitis |
| M86.3 | Chronic multifocal osteomyelitis |
| M86.4 | Chronic osteomyelitis with draining sinus |
| M86.5 | Other chronic haematogenous osteomyelitis |
| M86.6 | Other chronic osteomyelitis |
| M86.8 | Other osteomyelitis |
| M86.9 | Osteomyelitis, unspecified |
| M90.1 | Periostitis in other infectious diseases classified elsewhere |
| N10 | Acute tubulo-interstitial nephritis |
| N12 | Tubulo-interstitial nephritis, not specified as acute or chronic |
| N13.6 | Pyonephrosis |
| N15.1 | Renal and perinephric abscess |
| N30.0 | Acute cystitis |
| N30.8 | Other cystitis |
| N30.9 | Cystitis, unspecified |
| N34.0 | Urethral abscess |
| N39.0 | Urinary tract infection, site not specified |
| N41.0 | Acute prostatitis |
| N41.2 | Abscess of prostate |
| N41.3 | Prostatocystitis |
| N41.8 | Other inflammatory diseases of prostate |
| N41.9 | Inflammatory disease of prostate, unspecified |
| N43.1 | Infected hydrocele |
| N45.0 | Orchitis, epididymitis and epididymo-orchitis with abscess |
| N45.9 | Orchitis, epididymitis and epididymo-orchitis without abscess |
| N48.1 | Balanoposthitis |
| N48.2 | Other inflammatory disorders of penis |
| N49.0 | Inflammatory disorders of seminal vesicle |
| N49.1 | Inflammatory disorders of spermatic cord, tunica vaginalis and vas deferens |
| N49.2 | Inflammatory disorders of scrotum |
| N49.8 | Inflammatory disorders of other specified male genital organs |
| N49.9 | Inflammatory disorder of unspecified male genital organ |
| N51.0 | Disorders of prostate in diseases classified elsewhere |
| N51.1 | Disorders of testis and epididymis in diseases classified elsewhere |
| N61 | Inflammatory disorders of breast |
| N70.0 | Acute salpingitis and oophoritis |
| N70.1 | Chronic salpingitis and oophoritis |
| N70.9 | Salpingitis and oophoritis, unspecified |
| N71.0 | Acute inflammatory disease of uterus |
| N71.9 | Inflammatory disease of uterus, unspecified |
| N72 | Inflammatory disease of cervix uteri |
| N73.0 | Acute parametritis and pelvic cellulitis |
| N73.1 | Chronic parametritis and pelvic cellulitis |
| N73.2 | Unspecified parametritis and pelvic cellulitis |
| N73.3 | Female acute pelvic peritonitis |
| N73.5 | Female pelvic peritonitis, unspecified |
| N73.9 | Female pelvic inflammatory disease, unspecified |
| N75.1 | Abscess of Bartholin's gland |
| N76.0 | Acute vaginitis |
| N76.1 | Subacute and chronic vaginitis |
| N76.2 | Acute vulvitis |
| N76.3 | Subacute and chronic vulvitis |
| N76.4 | Abscess of vulva |
| N76.8 | Other specified inflammation of vagina and vulva |
| O03.0 | Spontaneous abortion, incomplete, complicated by genital tract and pelvic infection |
| O03.5 | Spontaneous abortion, complete or unspecified, complicated by genital tract and pelvic infection |
| O04.0 | Medical abortion, incomplete, complicated by genital tract and pelvic infection |
| O04.5 | Medical abortion, complete or unspecified, complicated by genital tract and pelvic infection |
| O05.0 | Other abortion, incomplete, complicated by genital tract and pelvic infection |
| O05.5 | Other abortion, complete or unspecified, complicated by genital tract and pelvic infection |
| O06.0 | Unspecified abortion, incomplete, complicated by genital tract and pelvic infection |
| O06.5 | Unspecified abortion, complete or unspecified, complicated by genital tract and pelvic infection |
| O07.0 | Failed medical abortion, complicated by genital tract and pelvic infection |
| O07.5 | Other and unspecified failed attempted abortion, complicated by genital tract and pelvic infection |
| O08.0 | Genital tract and pelvic infection following abortion and ectopic and molar pregnancy |
| O23.0 | Infections of kidney in pregnancy |
| O23.1 | Infections of bladder in pregnancy |
| O23.2 | Infections of urethra in pregnancy |
| O23.3 | Infections of other parts of urinary tract in pregnancy |
| O23.4 | Unspecified infection of urinary tract in pregnancy |
| O23.5 | Infections of the genital tract in pregnancy |
| O23.9 | Other and unspecified genitourinary tract infection in pregnancy |
| O41.1 | Infection of amniotic sac and membranes |
| O86.1 | Other infection of genital tract following delivery |
| O86.2 | Urinary tract infection following delivery |
| O86.3 | Other genitourinary tract infections following delivery |
| O86.4 | Pyrexia of unknown origin following delivery |
| O91.0 | Infection of nipple associated with childbirth |
| O91.1 | Abscess of breast associated with childbirth |
| O91.2 | Nonpurulent mastitis associated with childbirth |
| O98.2 | Gonorrhoea complicating pregnancy, childbirth and the puerperium |
| O98.8 | Other maternal infectious and parasitic diseases complicating pregnancy, childbirth and the puerperium |
| O98.9 | Unspecified maternal infectious or parasitic disease complicating pregnancy, childbirth and the puerperium |
| P36.0 | Sepsis of newborn due to streptococcus, group B |
| P36.1 | Sepsis of newborn due to other and unspecified streptococci |
| P36.2 | Sepsis of newborn due to Staphylococcus aureus |
| P36.3 | Sepsis of newborn due to other and unspecified staphylococci |
| P36.4 | Sepsis of newborn due to Escherichia coli |
| P36.5 | Sepsis of newborn due to anaerobes |
| P36.8 | Other bacterial sepsis of newborn |
| P36.9 | Bacterial sepsis of newborn, unspecified |
| P38 | Omphalitis of newborn with or without mild haemorrhage |
| P39.0 | Neonatal infective mastitis |
| P39.2 | Intra-amniotic infection of fetus, not elsewhere classified |
| P39.3 | Neonatal urinary tract infection |
| P39.4 | Neonatal skin infection |
| P39.8 | Other specified infections specific to the perinatal period |
| P39.9 | Infection specific to the perinatal period, unspecified |
| P77 | Necrotizing enterocolitis of fetus and newborn |
| R02 | Gangrene, not elsewhere classified |
| R57.8 | Other shock |
| T80.2 | Infections following infusion, transfusion and therapeutic injection |
| T81.4 | Infection following a procedure, not elsewhere classified |
| T82.6 | Infection and inflammatory reaction due to cardiac valve prosthesis |
| T82.7 | Infection and inflammatory reaction due to other cardiac and vascular devices, implants and grafts |
| T83.5 | Infection and inflammatory reaction due to prosthetic device, implant and graft in urinary system |
| T83.6 | Infection and inflammatory reaction due to prosthetic device, implant and graft in genital tract |
| T84.5 | Infection and inflammatory reaction due to internal joint prosthesis |
| T84.6 | Infection and inflammatory reaction due to internal fixation device [any site] |
| T84.7 | Infection and inflammatory reaction due to other internal orthopaedic prosthetic devices, implants and grafts |
| T84.9 | Unspecified complication of internal orthopaedic prosthetic device, implant and graft |
| T85.7 | Infection and inflammatory reaction due to other internal prosthetic devices, implants and grafts |
| T87.4 | Infection of amputation stump |
| T88.0 | Infection following immunization |
| U04.9 | Agent resistant to penicillin and related antibiotics |
| U80.0 | Penicillin resistant agent |
| U80.1 | Methicillin resistant agent |
| U80.8 | Agent resistant to other penicillin-related antibiotic |
| U81.0 | Vancomycin resistant agent |
| U81.8 | Agent resistant to other vancomycin-related antibiotic |
| U88 | Agent resistant to multiple antibiotics |
| U89.8 | Agent resistant to other single specified antibiotic |
| U89.9 | Agent resistant to unspecified antibiotic |

1. Immunocompromised state codes

3.1 Diagnosis codes of immunocompromised state

| ICD–10 | Code description |
| --- | --- |
| B20.0 | HIV disease resulting in mycobacterial infection |
| B20.1 | HIV disease resulting in other bacterial infections |
| B20.2 | HIV disease resulting in cytomegaloviral disease |
| B20.3 | HIV disease resulting in other viral infections |
| B20.4 | HIV disease resulting in candidiasis |
| B20.5 | HIV disease resulting in other mycoses |
| B20.6 | HIV disease resulting in Pneumocystis carinii pneumonia |
| B20.7 | HIV disease resulting in multiple infections |
| B20.8 | HIV disease resulting in other infectious and parasitic diseases |
| B20.9 | HIV disease resulting in unspecified infectious or parasitic disease |
| B21.0 | HIV disease resulting in Kaposi's sarcoma |
| B21.1 | HIV disease resulting in Burkitt's lymphoma |
| B21.2 | HIV disease resulting in other types of non-Hodgkin's lymphoma |
| B21.3 | HIV disease resulting in other malignant neoplasms of lymphoid, haematopoietic and related tissue |
| B21.7 | HIV disease resulting in multiple malignant neoplasms |
| B21.8 | HIV disease resulting in other malignant neoplasms |
| B21.9 | HIV disease resulting in unspecified malignant neoplasm |
| B22.0 | HIV disease resulting in encephalopathy |
| B22.1 | HIV disease resulting in lymphoid interstitial pneumonitis |
| B22.2 | HIV disease resulting in wasting syndrome |
| B22.7 | HIV disease resulting in multiple diseases classified elsewhere |
| B23.1 | HIV disease resulting in (persistent) generalized lymphadenopathy |
| B23.2 | HIV disease resulting in haematological and immunological abnormalities, not elsewhere classified |
| B23.8 | HIV disease resulting in other specified conditions |
| B24 | Unspecified human immunodeficiency virus [HIV] disease |
| B59 | Pneumocystosis |
| D47.1 | Chronic myeloproliferative disease |
| D70 | Agranulocytosis |
| D71 | Functional disorders of polymorphonuclear neutrophils |
| D72.0 | Genetic anomalies of leukocytes |
| D80.0 | Hereditary hypogammaglobulinaemia |
| D80.1 | Nonfamilial hypogammaglobulinaemia |
| D80.2 | Selective deficiency of immunoglobulin A [IgA] |
| D80.3 | Selective deficiency of immunoglobulin G [IgG] subclasses |
| D80.4 | Selective deficiency of immunoglobulin M [IgM] |
| D80.5 | Immunodeficiency with increased immunoglobulin M [IgM] |
| D80.6 | Antibody deficiency with near-normal immunoglobulins or with hyperimmunoglobulinaemia |
| D80.7 | Transient hypogammaglobulinaemia of infancy |
| D80.8 | Other immunodeficiencies with predominantly antibody defects |
| D80.9 | Immunodeficiency with predominantly antibody defects, unspecified |
| D81.0 | Severe combined immunodeficiency [SCID] with reticular dysgenesis |
| D81.1 | Severe combined immunodeficiency [SCID] with low T- and B-cell numbers |
| D81.2 | Severe combined immunodeficiency [SCID] with low or normal B-cell numbers |
| D81.3 | Adenosine deaminase [ADA] deficiency |
| D81.4 | Nezelof's syndrome |
| D81.5 | Purine nucleoside phosphorylase [PNP] deficiency |
| D81.6 | Major histocompatibility complex class I deficiency |
| D81.7 | Major histocompatibility complex class II deficiency |
| D81.8 | Other combined immunodeficiencies |
| D81.9 | Combined immunodeficiency, unspecified |
| D82.0 | Wiskott-Aldrich syndrome |
| D82.1 | Di George's syndrome |
| D82.2 | Immunodeficiency with short-limbed stature |
| D82.3 | Immunodeficiency following hereditary defective response to Epstein-Barr virus |
| D82.4 | Hyperimmunoglobulin E [IgE] syndrome |
| D82.8 | Immunodeficiency associated with other specified major defects |
| D82.9 | Immunodeficiency associated with major defect, unspecified |
| D83.0 | Common variable immunodeficiency with predominant abnormalities of B-cell numbers and function |
| D83.1 | Common variable immunodeficiency with predominant immunoregulatory T-cell disorders |
| D83.2 | Common variable immunodeficiency with autoantibodies to B- or T-cells |
| D83.8 | Other common variable immunodeficiencies |
| D83.9 | Common variable immunodeficiency, unspecified |
| D84.0 | Lymphocyte function antigen–1 [LFA–1] defect |
| D84.1 | Defects in the complement system |
| D84.8 | Other specified immunodeficiencies |
| D84.9 | Immunodeficiency, unspecified |
| D89.8 | Other specified disorders involving the immune mechanism, not elsewhere classified |
| D89.9 | Disorder involving the immune mechanism, unspecified |
| E40 | Kwashiorkor |
| E41 | Nutritional marasmus |
| E42 | Marasmic kwashiorkor |
| E43 | Unspecified severe protein-energy malnutrition |
| I12.0 | Hypertensive renal disease with renal failure |
| I13.1 | Hypertensive heart and renal disease with renal failure |
| I13.2 | Hypertensive heart and renal disease with both (congestive) heart failure and renal failure |
| K91.2 | Postsurgical malabsorption, not elsewhere classified |
| N18.0 | End-stage renal disease |
| N18.8 | Other chronic renal failure |
| T86.0 | Bone-marrow transplant rejection |
| T86.1 | Kidney transplant failure and rejection |
| T86.2 | Heart transplant failure and rejection |
| T86.3 | Heart-lung transplant failure and rejection |
| T86.4 | Liver transplant failure and rejection |
| T86.8 | Failure and rejection of other transplanted organs and tissues |
| T86.9 | Failure and rejection of unspecified transplanted organ and tissue |
| Y83.0 | Surgical Operation with transplant of whole organ or tissue |
| Z49.0 | Preparatory care for dialysis |
| Z49.1 | Extracorporeal dialysis |
| Z49.2 | Other dialysis |
| Z94.0 | Kidney transplant status |
| Z94.1 | Heart transplant status |
| Z94.2 | Lung transplant status |
| Z94.3 | Heart and lungs transplant status |
| Z94.4 | Liver transplant status |
| Z94.8 | Other transplanted organ and tissue status |
| Z94.9 | Transplanted organ and tissue status, unspecified |

- 1. Procedure codes of immunocompromised state

| 13706-00 | Allo bm/sc trnsplnt rel don wo in vitro |
| --- | --- |
| 13706-06 | Allo bm/sc trnsplnt rel don w in vitro |
| 13706-07 | Autolgs bm/stem cel trnsplnt wo in Vitro |
| 13706-08 | Autolgs bm/stem cell trnsplnt w in vitro |
| 13706-09 | Allo bm/sc trnsplnt oth don wo in vitro |
| 13706-10 | Allo bm/sc trnsplnt oth don w in vitro |
| 14203-01 | Direct living tissue implantation |
| 36503-00 | Kidney transplantation |
| 90172-00 | Sequential single lung trnsplnt bil |
| 90172-01 | Other transplantation of lung |
| 90205-00 | Heart transplantation |
| 90205-01 | Heart and lung transplantation |
| 90317-00 | Transplantation of liver |
| 90324-00 | Transplantation of pancreas |

ICD-10-AM 8th edition
