# Supplementary material for: The impact of post-operative sepsis on mortality after hospital discharge among elective surgical patients: a population-based cohort study
Source: Crit Care. 2017 Feb 20;21:34. doi: 10.1186/s13054-016-1596-7 (PMC5319141; doi:10.1186/s13054-016-1596-7)
Supplement: Additional file 3: Appendix 3. — Procedure codes from ICD-10-AM for selected surgical procedures. (DOCX 15 kb) [file 13054_2016_1596_MOESM3_ESM.docx]

**Appendix 3 Procedure codes from ICD-10-AM for selected surgical procedures**

| Coronary artery bypass graft | 38497-00  38497-01  38497-02  38497-03  38497-04  38497-05  38497-06  38497-07  38500-00  38503-00  38500-01 | 38503-01  38500-02  38503-02  38500-03  38503-03  38500-04  38503-04  90201-00  90201-01  90201-02  90201-03 |
| --- | --- | --- |
| Abdominal surgery | See reference [24] |  |
| EVAR | 33116-00 |  |
| Total hip replacement | 49318-00  49319-00  49324-00  49327-00 | 49330-00  49333-00  49345-00 |
| Total knee replacement | 49518-00  49519-00  49521-00  49521-01  49521-02  49521-03  49524-00 | 49524-01  49534-00  49530-00  49530-01  49533-00  49554-00  49527-00 |

EVAR= endovascular aneurysm repair
