# Supplementary material for: The impact of post-operative sepsis on mortality after hospital discharge among elective surgical patients: a population-based cohort study
Source: Crit Care. 2017 Feb 20;21:34. doi: 10.1186/s13054-016-1596-7 (PMC5319141; doi:10.1186/s13054-016-1596-7)
Supplement: Additional file 4: Appendix 4. — Results from sensitivity analyses. (DOCX 20 kb) [file 13054_2016_1596_MOESM4_ESM.docx]

**Appendix 4 Results from sensitivity analyses**

**Table A: The treatment effect of in hospital sepsis on post-discharge mortality (risk difference: results of nearest neighbour matching (NNM) and propensity score matching (PSM)**

| Post-discharge mortality (%) | Nearest neighbour matching(NNM; n=124,591) | | Propensity score matching (PSM; n=144,332) | |
| --- | --- | --- | --- | --- |
|  | Treatment effect ( %: Sepsis vs non-sepsis) | 95%CI of the treatment effect | Treatment effect ( %: Sepsis vs non-sepsis) | 95%CI of the treatment effect |
| 3-month | 2.8% | (1.3% - 4.3%)*** | 2.2% | (0.3% - 4.1%)* |
| 6-month | 3.8% | (2.3% - 5.3%)*** | 3.4% | (0.9% - 5.9%)** |
| 1-year | 5.4% | (3.0% - 7.7%)*** | 5.3% | (2.3% - 8.2%)** |

*p<0.05; **p<0.01; ***p<0.001

Note: 1) For NNM, the matching was based on admission year, age, gender, country of birth, marital status, comorbidity (Charlson Index), social-economic status (quartile of SEIFA score), location of the hospital (Rural/regional vs others), hospital peer groups, surgical type and length of stay (the exact matching was made on age groups, country of birth and comorbidity). The estimation of treatment effect was based on robust variance estimator and adjusted for age and length of stay. For PSM, same matching variables were used except that there were no exact matching groups and no adjustment for the continuous variables of age and length of stay.

2) For both NNM and PSM, a caliper of 0.05 was employed and a check of overlap of both baseline distribution and matched-sample were examined. Both graphic plots and standardised statistical summary and tests were employed wherever appropriate to make sure the balance of matched-sample as part of the iterative process.
